# A Novel Methodology for Characterizing Cell Subpopulations in Automated Time-lapse Microscopy

**DOI:** 10.3389/fbioe.2018.00017

**Published:** 2018-02-28

**Authors:** Georges Hattab, Veit Wiesmann, Anke Becker, Tamara Munzner, Tim W. Nattkemper

**Affiliations:** ^1^Faculty of Technology, Int. Research Training Group 1906, Computational Methods for the Analysis of the Diversity and Dynamics of Genomes (DiDy), Bielefeld University, Bielefeld, Germany; ^2^Faculty of Technology, Biodata Mining Group, Bielefeld University, Bielefeld, Germany; ^3^Department of Image Processing and Medical Engineering, Fraunhofer-Institut für Integrierte Schaltungen (IIS), Erlangen, Germany; ^4^SYNMIKRO, Phillips-Universität Marburg, LOEWE-Centre for Synthetic Microbiology, Marburg, Germany; ^5^Department of Computer Science, University of British Columbia, Vancouver, BC, Canada

**Keywords:** bioimaging, bioimage informatics, cell lineage, bacteria, microfluidics, synthetic biology, image processing

## Abstract

Time-lapse imaging of cell colonies in microfluidic chambers provides time series of bioimages, i.e., biomovies. They show the behavior of cells over time under controlled conditions. One of the main remaining bottlenecks in this area of research is the analysis of experimental data and the extraction of cell growth characteristics, such as lineage information. The extraction of the cell line by human observers is time-consuming and error-prone. Previously proposed methods often fail because of their reliance on the accurate detection of a single cell, which is not possible for high density, high diversity of cell shapes and numbers, and high-resolution images with high noise. Our task is to characterize subpopulations in biomovies. In order to shift the analysis of the data from individual cell level to cellular groups with similar fluorescence or even subpopulations, we propose to represent the cells by two new abstractions: the particle and the patch. We use a three-step framework: preprocessing, particle tracking, and construction of the patch lineage. First, preprocessing improves the signal-to-noise ratio and spatially aligns the biomovie frames. Second, cell sampling is performed by assuming particles, which represent a part of a cell, cell or group of contiguous cells in space. Particle analysis includes the following: particle tracking, trajectory linking, filtering, and color information, respectively. Particle tracking consists of following the spatiotemporal position of a particle and gives rise to coherent particle trajectories over time. Typical tracking problems may occur (e.g., appearance or disappearance of cells, spurious artifacts). They are effectively processed using trajectory linking and filtering. Third, the construction of the patch lineage consists in joining particle trajectories that share common attributes (i.e., proximity and fluorescence intensity) and feature common ancestry. This step is based on patch finding, patching trajectory propagation, patch splitting, and patch merging. The main idea is to group together the trajectories of particles in order to gain spatial coherence. The final result of CYCASP is the complete graph of the patch lineage. Finally, the graph encodes the temporal and spatial coherence of the development of cellular colonies. We present results showing a computation time of less than 5 min for biomovies and simulated films. The method, presented here, allowed for the separation of colonies into subpopulations and allowed us to interpret the growth of colonies in a timely manner.

## 1 Introduction

A bacterial cell colony is a group of bacteria grown from a single parent cell on a culture or medium. The capture of colony growth is possible thanks to time-lapse imaging, in particular high-resolution microscopy. Temporal and spatial changes are recorded to provide detailed information on the growth of cell colonies. Ultimately, the analysis of these records leads to characterization of cellular behavior at different scales of biological organization (i.e. cell, subpopulation, and colony). These spatiotemporal records comprise a sequence of digital microscopy images, referred to in this article as “biomovies”. The combination of such biomovies with genetically modified reporter gene fusions allows the detection of changes in gene expression by changes in cell fluorescence, as can be seen in other works pertaining to antibiotic resistance (Sun et al., 2011; Mohan et al., 2013).

In the context of this work, we examine biomovies that show the growth of the *Sinorhizobium meliloti* bacterium. The resulting biomovies help us to study its gene regulation and phenotypic heterogeneity under stressful conditions (Charoenpanich et al., 2015; Schlüter et al., 2015a). Our goal is to gain a better understanding of the patterns emerging within the colony, by locally finding subpopulations of cells with similar fluorescence patterns over time and space. Fluorescence intensities were measured according to Schlüter et al. (2015a) and are hence comparable across frames. A complete experiment consists of multiple conditions, each of which is recorded as an individual biomovie.

**Figure 1:**
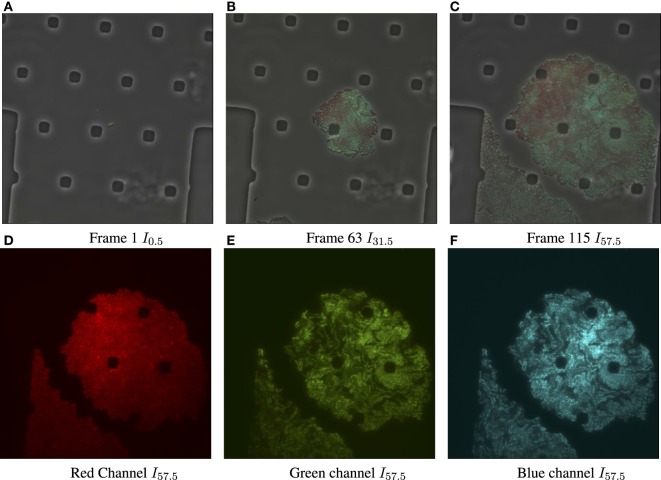
Input data for biomovie D1, with exposure set to 100%. **(A)** Original image frame in RGB color space overlaying the luminance channel (phase contrast image) at the first time point. We observe a set of particular and square-like polygons. They are an intrinsic part of the microfluidic chamber, in which bacteria grows. **(B)** Time 31.5 h. **(C)** Time 57.5 h, the final frame. **(D)** The dissociated red channel for the final frame. **(E)** Green channel. **(F)** Blue channel.

The general paradigm for the analysis of such data is centered on the extraction of information from the cell lineage of all visible cells, for example in the studies by Schneider et al. (2012) and Helfrich et al. (2015), which ultimately leads to its visualization, as described in the study by Pretorius et al. (2016). A cell lineage is a sequence of cells that have developed from a common ancestor. This extraction step includes the segmentation of single cells, their tracking, and the lineage construction. Segmentation refers to spatial coherence and involves delineating individual cells in each frame. Tracking refers to temporal coherence and involves the monitoring of cells throughout a biomovie. Lineage construction is meant to identify cell division events, also referred to as the correspondence problem to trace cellular ancestry (see Figure S1 in Supplementary Material). However, the extraction of cell lineages from microfluidic biomovies such as the one shown in Figure 1 is a challenge due to the high cell count (~300), considerable variation in cell size and shape, high cell density and a strong noise, and low temporal resolution (1 frame/30 min). The time-lapse studies presented herein are based on high-resolution microscopy with the 2,000 nm limit, where the pixel size is less than the optical resolution. When colonies have high cell density, even rod-shaped and anisotropic bacterial cells may appear to have different shapes due to contact between cells. The inadequacy of automatic methods for data with such characteristics led experts in the field to a manual annotation process. It is extremely time-consuming, arduous, and error-prone in terms of low intra- and inter-observer agreement. Our collaborators need a period of about two to three working days to annotate a biomovie and create a bacterial cell lineage. Furthermore, the comparison of data sets/biomovies between different experiments justifies the need for better computational support and automatic lineage extraction approaches.

Related work for the computational analysis of biomovies is summarized in Table 1. None of these approaches have been successful in extracting lineage information from the data considered here (i.e., biomovies). The inability to process these biomovies was attributed to high values for different data properties. The five data properties are as follows: cell count, cell shape diversity, cell density, noise, and lateral resolution (60 nm/px). In addition, the large time separation between images (i.e., temporal resolution) presents one of the greatest challenges in cell tracking.

**Table 1:** Related work is cataloged according to cell type and colony properties.

	Colony properties/related work	Cell count	Cell shape diversity	Cell density	Noise	Resolution (nm/px)	Species
Prokaryotic	Klein et al. (2012)	Moderate (~100)	Low	Low	Low	NS	*B. megaterium*
	Mekterović et al. (2014)	Low (~30)	High	High	Low	Moderate (129)	*M. smegmatis*
	Grunberger et al. (2015)^a,b^	Low (~50)	Moderate	Moderate	Low	Low–moderate (< 120)	*C. glutamicum*

Eukaryotic	Kanade et al. (2011)^b^	High (>200)	Low	Low	High	Moderate (130)	*B. taurus*
	Bao et al. (2006)	High (350)	Low	Low	Moderate	Moderate (100)	*C. elegans*
	Li et al. (2008)^b^	Very high (>500)	High	High	High	Moderate (130)	*H. sapiens*

Both	Wang et al. (2005)	Low	Low	Low	Low	NS	*E. coli* and *H. sapiens*

*^a^Larger colonies did not have their lineage constructed*.

*^b^Approach or framework is not available*.

*NS, not stated in the publication*.

Our view postulates that the shortcomings of previous methods are due to the general paradigm of analysis (i.e., single-cell oriented). As described earlier, these methods rely on an initial segmentation step before continuing with tracking and lineage construction. The closest relevant effort was reported in the study by Grunberger et al. (2015). However, instead of computing single cell lineages for large and medium-size experiments, the cell area of interest is quantified by calculating its logarithm.

In this work, our primary objective is to characterize different cell behaviors within a colony that are consistent in space and time (i.e., coherent subpopulations). As a task, the search for bacterial subpopulations is linked to many biological questions, such as bacterial pathogenesis (David, 2013) or the study of metabolic interactions (Rosenthal et al., 2017). Addressing one of these problems from a different biological scale could provide a different perspective or even speed up data curation and interpretation. In this work, analysis of bacterial subpopulations may relate either to the subset of the data (i.e., a biomovie) that was generated in a single experimental condition or to cellular attributes shared between all data (i.e., different biomovies) collected as part of the full experiment.

In light of the above-mentioned data properties, we propose an alternative to the single-cell oriented paradigm that combines spatial and temporal coherence. We are considering two new data abstractions that allow us to characterize cell subpopulations in biomovies. We are introducing a flexible modular framework to investigate changes in (C)olon(Y) growth and (C)ell (A)ttributes in (SP)atiotemporal experiments (CYCASP). This framework is designed to manage the dynamics of rapid growth and the diversity of bacterial forms using two new abstractions, which we call the *particle* and the *patch*. In this section, we give the general motivation behind these abstractions, with the formal definitions of particle and patches that follow in the Methods section.

A **particle** is a geometric abstraction that results from taking into account the fact that the neighborhood around a pixel falls into a cell by checking for signal characteristics such as signal intensity, edge orientation, fluorescence signals, or texture. A **particle trajectory** is assembled by tracking a particle over time, exploiting temporal coherence to filter out interfering or spurious signals that do not persist over multiple frames. A **patch** is the aggregation of spatially contiguous particles that feature similar signal characteristics. A **patch trajectory** reflects the evolution of patches over several frames. Therefore, it is the aggregation of similar particle trajectories and represents cell subpopulations with similar fluorescence profiles. Finally, a **patch lineage** encapsulates the splitting and joining of all patch trajectories that descend from a common ancestor. Although a cell lineage is clearly a tree rooted from an ancestor cell that divides into its descendants as the colony grows, a patch lineage is in fact a directed acyclic graph (DAG). Smaller patches of similar fluorescence that are spatially separated in an earlier frame may eventually merge together into one larger patch in a later frame. This occurs as cells continue to divide and react to their environment: Biomovies with larger colonies (>100 individuals) can contain multiple patch lineages from multiple ancestor cells.

Due to the high values for the fives properties and the low temporal resolution, it is not possible to segment and track individual cells. Our approach opts for groups of particle trajectories in order to obtain a better granularity of the colony’s growth. It allows us to process both identification and tracking of bacterial subpopulations. Contrary to a minimum of 2 days of manual analyses previously required of our collaborators, our reference results show that CYCASP can automatically extract patch lineages from biomovies in less than 5 min for biological data sets of more than 100 frames and 300 cells. We also discuss the parameters needed to properly track particles through space and time and aggregate them into patches. The CYCASP framework for extracting coherent lineages for entire cell colonies is available free of charge at https://github.com/ghattab/cycasp.

## 2 Materials

Four biomovies and five simulated movies are used in this paper. The simulation system that created the simulated movies is reported in the supporting information. Table S1 in Supplementary Material contains a detailed description of biomovies and simulated movies. The bioimaging system that created the biological data is described below.

The four biomovies D1–D4 were acquired by coupling phase contrast microscopy and total internal reflection fluorescence (TIRF) microscopy with a frequency every 30 min (temporal resolution) using a TIRF laser at a lateral resolution of 60 nm/px. Given this low temporal resolution and the exponential bacterial growth, biomovies D1–D4 are particularly complex. The output is four 1,004 × 1,002 px images per channel (luminance and RGB) in uncompressed TIF format. All microcolonies grew on a flat plane between two membranes that fit onto the microwell plate of the microfluidic device. This membrane prevents bacterial cells from overlapping. Two experiments E1 and E2 were carried out to record four data sets (D1, D2 from E1 and D3, D4 from E2). In the first experiment (E1), the heterogeneity of a particular promoter is monitored (Schlüter et al., 2015b). This promoter is responsible for the expression state of the galactoglucan biosynthesis gene group. To express this exopolysaccharide, two copies are used: one fused with cerulean and one with mVenus coding regions, representing in turn the state of this gene group. For this experiment two biomovies result: D1 and D2. In the second experiment (E2), the objective is to understand the behavior of colonies and other phenomena such as the detection of quorum sensing (McIntosh and Bettenworth, 2017). The activity of a promoter representing the cell’s quorum status is monitored. This promoter is merged with the mVenus coding region, in addition to monitoring the state of activity of one of the above-mentioned promoters, which is merged with the cerulean coding region. In each experiment, the constitutive T5 promoter fused to the mCherry coding region was used as a marker to label viable and metabolic active cells. This results into two other biomovies: D3 and D4.

In both experiments, each cell in the isogenic bacterial population under investigation is fluorescing differently in the RGB channels. Fluorescence changes reflect changes in the state of cells. They are mediated by promoter–reporter gene fusions and are triggered by various factors. These include stochastic effects, adaptation to environmental conditions such as diffusible signals, nutrient availability, or other unknown factors. For E1, bacterial cells exhibit an active (constitutive) fluorescence in the red channel (i.e., expressing the mCherry protein). However, this is the case with the green channel for E2. Once a bacterium undergoes changes in the expression of the monitored genes, the fluorescence profile changes from red exclusively to yellow-green. Theoretically, no fluorescence indicates that the cell is likely dead or in a persistent state with very low metabolic activity. Practically, even a cell line with stable expression might not show fluorescence for a plethora of reasons. This particular shortcoming is managed by the presented framework (see Particle trajectory linking section: time interval for (dis-)appearing particles). In both experiments, spatial information is crucial for identifying and locating nearby regions with similar intensity patterns.

In order to have a test data set with a structure similar to that of the experimental data D1–D4, we are extending a previously proposed cell simulation software for the computation of simulated cell colony movies (DS1–DS5) (Wiesmann et al., 2013). Simulated movies can be downloaded from the study of Wiesmann et al. (2017). The bacterial cell shapes are modeled as ellipses with a texture computed by a sigmoid function. In addition, cell positions are determined on a frame-by-frame basis by an energy minimization approach. Appendix S2 provides more details on the construction of simulated movies. In this manuscript, we refer to each image of the biomovie with *I_t_* where *t* is the time index (in hours).

In reference to our approach, the first author conducted a manual annotation for different frames of each biomovie and compared the number of annotated cells to the number of particles (see Figure S15 in Supplementary Material). The task of manual annotation was carried out using the professional annotation software BIIGLE 2.0 (Langenkämper et al., 2017). Due to the difficulty of the task, only one frame was annotated five times, as seen in Figure S16 in Supplementary Material. We report the results in the beginning of the Results section.

## 3 Methods

The CYCASP method processes a biomovie and a simulated movie in three consecutive steps: preprocessing, particle analysis, and patch trajectory computation. Figure S2 in Supplementary Material provides an overview of this framework.

### 3.1 Preprocessing

A pipeline of standard image processing steps is applied to the RGB channels of each frame *I_t_* to reduce noise, enhance the object-to-background contrast, and spatially align the images (Hattab et al., 2017). The output is a binary image $\hat{I_{t}^{\;}}$ for each time point. We use the RGB images since they are relatively less noisy than the phase contrast images. Pipeline details are provided in supporting Appendix S2.

### 3.2 Particle Analysis

In the second step described below, particles are detected and assembled into temporally coherent particle trajectories. The motivation consists mainly of characterizing changes in cellular attributes over time and finding consistent particle positions over time. We justify our focus on the temporal coherence of particles due to the low temporal resolution and uncertainty of cellular positions. Our approach allows particles to appear and disappear. In addition, the parameterization of the patch lineage algorithm allows us to obtain less or more stringent differences in fluorescence signals. Therefore, the main weight is on the temporal dimension. Our goal is to have enough particles to ensure that there are no false negatives (i.e., each cell is represented by at least one particle). False positives are detected and filtered using the parameter windows described below.

#### 3.2.1 Particle Detection

**Figure 2:**
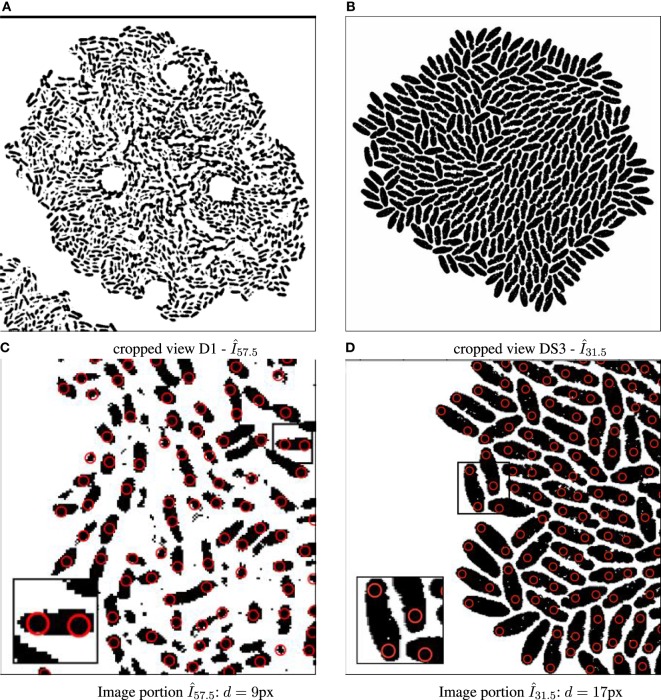
Binary images annotated with computed particle positions (shown as red circles). **(A)** Original biomovie D1 binary image. **(B)** Simulated movie binary image. **(C)** Original biomovie crop of D1 showing 1–2 particles detected within each cell. A particle diameter value of *d* = 9 px yields no false negatives, and some false positives that will be eliminated in subsequent processing that exploits temporal coherence. **(D)** Simulated movie crop showing ~2 particles detected per cell, with a particle diameter *d* = 17 px.

Although there are different avenues, such as the use of nanoparticles to facilitate cell tracking in automated time-lapse microscopy (Najafzadeh et al., 2016; Singh et al., 2017), this framework is the first to use “virtual” particles instead of individual cells to bypass the segmentation problem. Fundamentally, a nanoparticle is investigated as a carrier for intracellular tracking and drug delivery. When employed as a single fluorescent nanoparticle, it is often localized and followed in living cells using an imaging modality (Gardini et al., 2015). In contrast, the “virtual” particle is defined at the image space. In this case, the particle detection uses a Gaussian blob operator (Crocker and Grier, 1996; Allan et al., 2015) for each binary image at each time point for cells with a given expected diameter *d* (here set to *d* = 11 px, see illustration in Figure 2).

Each detected particle can be inscribed within a cell or group of contiguous cells. To treat anisotropic bacterial forms, we suggest calculating *d* based on the size of bacterial cells in the image space, where *l* = average bacterial length and *w* = average bacterial width (and *d* is odd):

(1) $$d=\{\begin{array}{cc} \mathrm{floor}(\max(l,w),\min(l,w),\frac{1}{2}*(\max(l,w)-\min(l,w))) & \text{if }l\neq w\\ \mathrm{floor}(w,\frac{1}{2}*w) & \text{else} \end{array}$$

Diameter size has an important effect on precision. If a user underestimates the particle diameter, the precision suffers. Therefore, it is preferable to overestimate the diameter, although larger diameters come at some cost in performance (Allan et al., 2015). Moreover, too small a diameter often tends to skew the location of a particle toward the pixel edges.

Often, a particle has visually distinct qualities, features, or attributes. They range from the spatial position of a particle to the integrated color information in the RGB domain. These features are introduced once particles are tracked over time.

#### 3.2.2 Particle Trajectories

A particle trajectory is assembled by following a particle in time, exploiting temporal coherence. This filters out spurious signals that do not persist over multiple frames. The life cycle of a particle, that is, the changes induced over time, ranges from creation, bifurcation, continuation, and dissipation to amalgamation (Ji et al., 2003). Particle tracking is used between consecutive frames, throughout the biomovie, on particles found with the Crocker and Grier’s algorithm (Crocker and Grier, 1996). The Python implementation of the algorithm is used: trackpy (Allan et al., 2015). All particle positions are evaluated across space and time using trajectory linking and filtering, respectively.

##### 3.2.2.1 Particle Trajectory Linking

To link particle positions (*x, y*)*_t_* into particle trajectories {*J_k_*}, we use the KDTree neighbor-finding strategy is employed (default method of trackpy) with the two parameter windows of distance and time. The distance radius *σ*_max_ = *d* − 2 px determines the maximum distance each particle is allowed to move from the initial position between consecutive images. The size of the time interval *W*_max_ = floor (15% frame count) determines the maximum number of consecutive images to be taken into account for (dis-)appearing particles. Particle trajectories {*J_k_*} are defined as:

(2) $$\text{\{}J_{k}\text{\}}=\text{\{}{\left(x,y\right)}_{t,p}\text{\}}$$

with 1 ≤ *k* ≤ *K* where *K* = number of particle trajectories and *p* = particle index. Particle trajectories are disjunct: if (*x, y*)*_t,p_* ∈ *J_k_* then

(3) $${\left(x,y\right)}_{t,p}\notin J_{k^{\prime }}\;\forall k\neq k^{\prime }.$$

##### 3.2.2.2 Particle Trajectory Filtering

Spurious trajectories are filtered out according to a time window *W*_min_ = floor (10% frame count). If (*x, y*)*_t_*_,_*_p_* ∈ *J_k_* with *t* = *t*_max_ < *W*_min_ then *J_k_* is omitted. Otherwise, the algorithm finds no spurious trajectories and continues onto the next computation.

##### 3.2.2.3 Particle Trajectory Color Information

A particle trajectory is reassociated with its underlying color information by extracting fluorescence values from the RGB channels at the given particle positions. RGB values are referred to with (*r_x,y_, g_x,y_, b_x,y_*)*_t,p_* and are normalized linearly based on the minimum and maximum values in each channel and across all images. The resulting RGB values are within the bounded range [0, 255], normalized to decrease low fluorescence and intensify high fluorescence signals. We maintain that the minimum value corresponds to either noise artifacts or spurious trajectories (dying cells that may prove difficult to follow). In this way, we filter out completely black particles.

### 3.3 Patch Lineages

To move from a particle level to a level of subpopulations, particle trajectories {*J_k_*} are processed in order to first identify patches, i.e., neighboring groups of particles with similar fluorescence behavior and then to compute trajectories for these patches.

#### 3.3.1 Algorithm Overview

**Figure 3:**
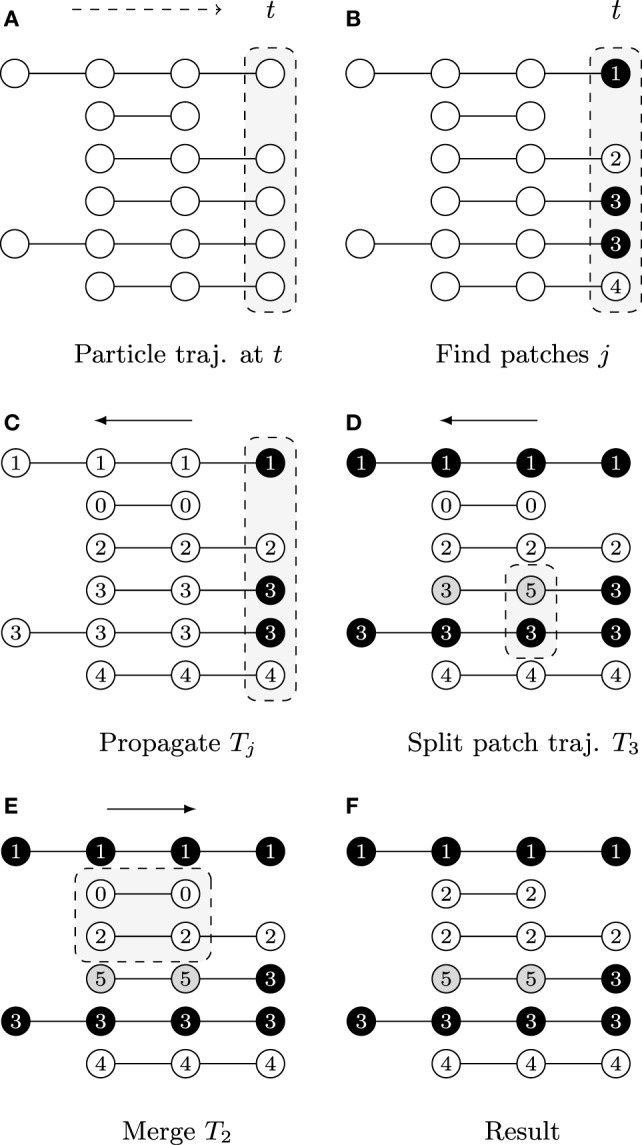
A graphical description illustrating the patch lineage construction algorithm, where each row shows a temporally coherent particle trajectory that is close to those above and below it in feature space. The dots represent particle positions at each time point and their coloring of white/gray/black represents differences found in feature space provided the user-specified thresholds. The slice of space-time that is the focus of computation in each subfigure is highlighted by gray boxes with dashed outlines. **(A)** Biomovies have a naturally occurring temporal direction, represented as a dashed arrow ending at time *t*. The trajectories have a different number of particles, showing that particles can appear at any time point. **(B)** Particle trajectories are grouped into patches at the last time point. **(C)** The trajectory information is propagated upstream in a run from the last to the first time point. **(D)** The split propagation proceeds from the last to the first time point. **(E)** The merge propagation runs from first to the last time point, mirroring biological growth. **(F)** The resulting patch lineage contains 5 patches.

The first step is achieved by evaluating heuristically defined constraints on feature vectors **v***_t_*_,_*_p_* of particles (*x, y*)*_t,p_*. The second step requires a split-and-merge procedure applied to the patch trajectories {*J_k_*}, as shown in Figure 3.

The first step is computed at the last time point, i.e., the biomovie’s last frame, and then the patch information is propagated upstream to earlier time points, rewinding to *t* = 0. The motivation for starting the computation from the last time point is biological: the maximum number of cells appears at the end of the growth sequence. The second step begins with the split procedure, which can be interpreted as intrapatch verification. This is also done at every time point from last to first. Positions are checked at every time point for all particles associated with a patch using the same patch finding approach as the first step. In the case of divergence, particles are split into a new patch. The final step ends with the merge procedure, i.e., the second step. The merge procedure performs an interpatch verification, from the first to the last time point, to determine whether every possible patch pair should merge or remain separate. The resulting patch trajectories reflect spatial and temporal coherence.

#### 3.3.2 Patch Finding

A patch at time point *t* is the aggregation of spatially contiguous particle trajectories that feature similar signal characteristics; that is, cell subpopulations with similar fluorescence patterns. To create patches from particles, in an image *I_t_*, we define a decision function Φ(**v***_t,p_*, **v***_t,p_*_′_) for the similarity in signal characteristics in the feature space of particles *p* and *p*′. With the feature vector **v***_t,p_*, defined as **v***_t,p_* = (*x_t,p_, y_t,p_, r_t,p_, g_t,p_, b_t,p_*). Φ could either be one Minkowski metric or a scalar product, joining together multiple particles into a coherent patch. For instance, in some cases, only one color channel might be considered (see Patch lineage graphs).

We are considering features from different domains, i.e., space (*x, y*) and color (*r, g, b*). The decision function Φ(*p, p*′) = {1, 0} is defined as a Boolean evaluation of different user thresholds, such as:

(4) $$\Phi (p,p^{\prime })=\Phi (v_{t,p},v_{t,p^{\prime }})=\Pi_{j}\phi_{j}(v_{t,p},v_{t,p^{\prime }})=\phi_{1}\cdot \phi_{2}\cdot \phi_{3}\cdot \phi_{4}$$

(5) $$\phi_{1}(v_{t,p},v_{t,p^{\prime }})=\{\begin{array}{ll} 1 & \text{if d}((x,y),(x^{\prime },y^{\prime }))<t_{d}\\ 0 & \text{else} \end{array}$$

(6) $$\phi_{2}(v_{t,p},v_{t,p^{\prime }})=\{\begin{array}{ll} 1 & \text{if }\delta_{r}=|r-r^{\prime }|<t_{r}\\ 0 & \text{else} \end{array}$$

(7) $$\phi_{3}(v_{t,p},v_{t,p^{\prime }})=\{\begin{array}{ll} 1 & \text{if }\delta_{g}=|g-g^{\prime }|<t_{g}\\ 0 & \text{else} \end{array}$$

(8) $$\phi_{4}(v_{t,p},v_{t,p^{\prime }})=\{\begin{array}{ll} 1 & \text{if }\delta_{b}=|b-b^{\prime }|<t_{b}\\ 0 & \text{else} \end{array}$$

with user thresholds for space (i.e., distance) *t_d_* and color *t_r_, t_g_, t_b_*, respectively. In principle, other functions can be defined to the user’s requirements.

The graphical example in Figure 3 shows particle trajectories projected onto rows where time runs from left to right. Particles are colored white, gray, or black to illustrate differences in the feature space, i.e., particle of the same gray value have similar features (Φ(**v***_t,p_*, **v***_t,p′_*) = 1). The patch lineage computation begins with an initial patch finding propagation at the last time point, as shown in Figure 3A. Particles that meet the feature thresholds are grouped into four patches labeled with distinct patch IDs in Figure 3B, where patch 3 contains two neighboring and similar particles of the same black color.

#### 3.3.3 Patch Trajectory Propagation

After a patch is found in the previous step, the decision is propagated upstream by employing the temporal coherence of particle trajectories *J_k_* to patch trajectories *T_j_*. The algorithm marches backwards from time *t*_max_→*t*_0_, inspecting each particle trajectory that appears in the frame, and either propagating the patch ID from downstream for existing particle trajectories or assigning a new patch ID when a new particle trajectory is first encountered that has not yet been assigned to a patch. Figure 3C shows the result, where the patch trajectory in the second row that has no particle trajectory visible in the last time point has been assigned the patch ID 0.

#### 3.3.4 Patch Trajectory Splitting

A second propagation, which also runs from *t*_max_ to *t*_0_, verifies at any given time point whether to split a patch into several patches because the fluorescence behavior is not uniform for all the particles in the patch. That is, if the user specified distance and color thresholds are surpassed. Depending on the size of a patch, a split may correspond to an emerging behavior within a subpopulation. Figure 3D shows an example where patch 3 is split when a feature change is noticed at the second to last time point, and the particle trajectory is assigned a new patch ID 5. For computational efficiency, we maintain at all times the concave hull of each patch, stored in the format of a list of bounding particles, using a Delaunay tessellation.

#### 3.3.5 Patch Trajectory Merging

In this third propagation, patch trajectories are compared iteratively over time but now in the forward direction from the first time point *t*_0_ to *t*_max_. The direction of this final computation deliberately corresponds to the biology of patch growth, where previously separated regions touch due to the growth of new cells. The merge computation requires checking for intersections between all pairs of patches that exist at each time point. We accelerate it with a rapid initial intersection test between the oriented bounding rectangles to exclude pairs of patches that do not have geometric overlaps. We only evaluate the full set of bounding particles in cases of intersections, which can range from a single contact to the complete inclusion of one patch in another. Separating the splitting and merging procedures into separate sequential propagations follows a chunking strategy. In addition, it is not necessary to carefully adjust splits to avoid “over-segmentation” into too small patch trajectories, as these are taken into account in this subsequent merge propagation. Figure 3E shows an example of how particle trajectories that are absent at the last time point are treated. The second particle trajectory received the patch ID 0 in the propagation phase. It is now joined with the third trajectory as patch 2 because it falls within the merge window threshold *δ_t_*. The final set of five patches is listed by their patch ID in Figure 3F. The set of patch trajectories is defined as {*T_j_*} with 0 ≤ *j* ≤ *N*, the number of patch trajectories *N*.

### 3.4 Computational Performance

The computational efficiency of our framework is based on the processing of a much smaller number of image objects at each step in a much more detailed way. Once preprocessing is complete, particle detection is initiated: a number of *m* particles are extracted very efficiently using only spatial coherence. Particle linking follows, *k* particle trajectories are constructed to exploit temporal coherence. Finally, patch lineages are constructed: *j* patch trajectories are computed using a multi-propagation algorithm with bidirectional propagations. The three quantities generally obey *m* > > *k* > > *j*, typically multiple particles are detected within each cell, so the number of particles *m* is greater than the cell count *c* per at least a factor of 2. In the Results section, we report computational performance by calculating the average elapsed time of 100 runs of the preprocessing step and the particle step, respectively.

### 3.5 Parameter Space

**Figure 4:**
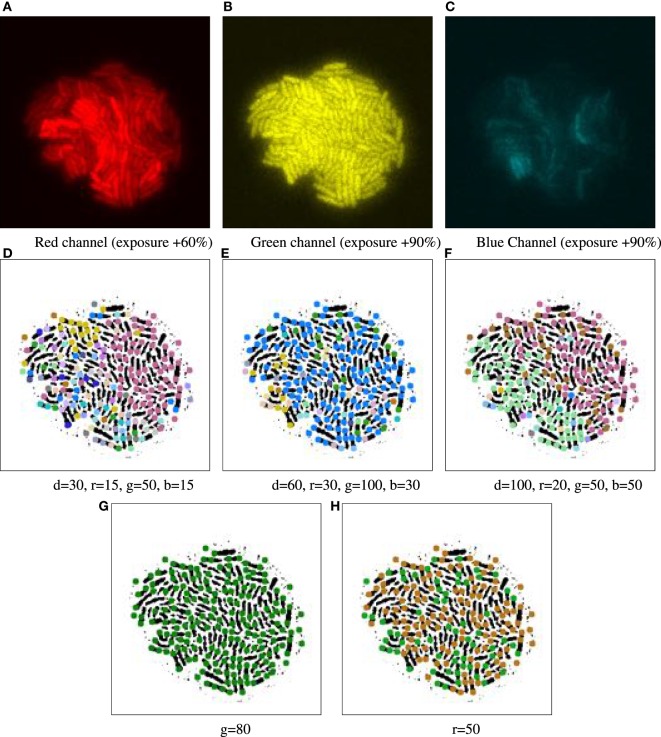
Example of parameter tuning to emphasize different channels, for time point 16.5 h of biomovie D3. The binary images in the bottom row are annotated with 9-px dots showing particle locations, colored according to their patch IDs. The particle analysis thresholds in the previous computational step were set to 9 px particle diameter, a 5 px distance and 10-frame window for particle linking and a 3-frame window for time filtering. **(A–C)** Separate views of red, green, and blue channels show the high structural variation between each channel. **(D–F)** Three different combinations of settings yield patch structures that capture different combinations of channel features, with thresholds for geometrical distance (d), and channel specific differences in (r, g, and b). **(G–H)** Two examples of sensitivity analysis for individual channel thresholds (G: green, H: red), where the other channels are ignored by setting appropriate thresholds (thresholds are set to very high values: geometric distance values near the total image size, and color values near the maximum of 255). **(G)** The threshold of 80 for green depicts a homogeneous and constant signal across that channel, yielding a single main patch. **(H)** The threshold of 50 for red emphasizes the binary nature of that signal, yielding two major patches. In both **(G)** and **(H)**, the observed patches are exempt of spatial contiguity due to excluding the spatial dimension.

Appropriate user thresholds *t_d_, t_r_, t_g_*, and *t_b_* have been determined for the feature vectors through empirical exploration. First, we examined the basic descriptive statistics of the biomovies in the geometric and color distance channels: the colony diameter in pixels, and for each color channel the minimum and maximum values, as well as the standard deviation. We then proceeded to the test phase by completely eliminating homogeneous channels to reduce the noise and using a sensitivity analysis for each feature threshold. This selective approach has allowed us to test the robustness of the results and to better understand the relationships between certain thresholds and a desired outcome. An example of an illustration is shown in Figure 4.

To avoid false negatives, i.e., cells not represented by any particle, we suggest using more particles than the number of cells, by at least a factor of 2. This is possible by setting the particle diameter *d* smaller than the minimum cell diameter (see Methods: Particle detection). There are two parameters that influence the linking of particle positions into trajectories: the distance radius *σ*_max_ and the time linking interval *W*_max_. The greater the distance radius is, the more particles are evaluated by the neighbor-finding strategy (see Particle detection). In addition, the larger the size of the time linking interval is, the greater the memory for particle positions in that time interval. As indicated in Methods: Particle trajectories, we have chosen reasonable values to limit computational expense. For trajectory filtering (time), it is reasonable to set the default filtering window based on the number of frames. Since short trajectories do not necessarily correspond to spurious ones, it is preferable to change the filtering window on a case-by-case basis. For example, under certain experimental conditions, cells may have a short life span.

## 4 Implementation

We implemented the CYCASP modular algorithm in Python. Specific packages were used, ranging from computer vision to tracking. The above-mentioned preprocessing step uses OpenCV (Bradski, 2000). The particle tracking step uses Trackpy (Allan et al., 2015), the tracking algorithm that provides functionality including static and motion analyses, particle position prediction and plotting tools. Mandatory packages are listed on https://github.com/ghattab/cycasp.

## 5 Results

As reported in the Materials section, we report below the results of the manual annotation. Observable cells were annotated per experiment and across the following frame numbers: 25, 35, 50, 75 and 10, 20, 30, 40 of biomovies (D1, D2) and (D3, D4), respectively (Figure S15 in Supplementary Material). To compare the number of annotated cells to the number of found particles (i.e., particle detection step), we calculated the ratio of found particles to annotated cells *r*. Although the particle detection step contains many parameters, they are set to the same values in each experiment and produce an identical trend (Figure S15 in Supplementary Material). In addition, when calculating regression models, we found that the particle concept demonstrated consistent robustness and trends for the E1 and E2 experiments with an average ratio of 1.8 and 2.4, respectively. Provided this ratio, we can estimate the number of cells, in biomovies D1 to D4.

Moreover, to test the intraobserver reliability, we selected time point 12.5 h or *t*_12.5_ of biomovie D2 and annotated the cells five times. We observed a variable number of cells or annotations from a minimum of 249 to a maximum of 300 (Figure S16 in Supplementary Material). The manual annotation results in a reliable intra-observer agreement with a mean μ = 267 and a standard deviation *σ* = 21.65.

We now present the results of applying CYCASP to four original data (D1–D4) and five simulated (DS1–DS5) movie data sets. The biological data sets present high values for the 5 properties targeted by this work: cell count, cell shape diversity, cell density, image noise, and image resolution. Table S1 in Supplementary Material provides all the details.

### 5.1 Preprocessing

Figure 2A shows the result of the preprocessing to improve the cell to background contrast in the RGB images shown in Figures 1C–F (frame *I*_115_ of D1). Figure S3, in Supporting Information, shows the results after each step of the preprocessing pipeline for the final frame of biomovie D1. The final binary images, after preprocessing the other biomovies and simulated movies, are shown in Figures S4–S8 in Supplementary Material.

#### 5.1.1 Computational Performance

The processing time corresponds to the number of frames in the biomovie, from a minimum of 6 s for the 25-frame simulated movie DS1 to a maximum of 53 s for the largest biomovie 115 frames biomovie D2. Figure S13 in Supplementary Material provides the performance details for all tested data sets.

### 5.2 Particle Analysis

We now evaluate the results of our particle analysis and the graphs of the patch lineage (in the next paragraph). This is accomplished at a technical level, in terms of success in capturing spatial coherence, temporal coherence, and computational performance. Next, we will discuss the biological interpretation of the results.

#### 5.2.1 Spatial Coherence

**Figure 5:**
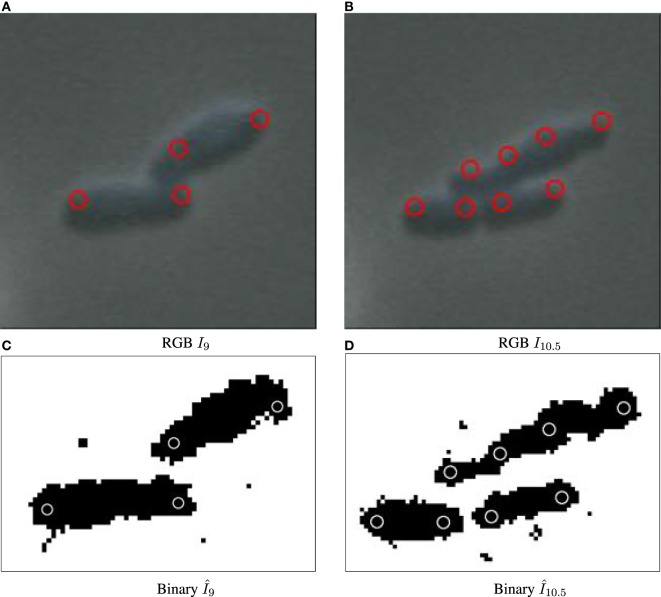
Particle detection for biomovie D1 across cell division events, with detected particle locations annotated as red circles on original images **(A–B)** and white circles on binary images **(C,D)**. The particle paradigm copes with cell division despite high levels of noise, and the direct contacts between cells: when the cell elongates, a new particle is created in the center when the width between the previous particles surpasses the distance threshold.

The particle approach used by CYCASP successfully captures the spatial coherence of cell subpopulations. Figures 5 and 2 show computed particle locations annotated as red circles on the RGB and the binary images, respectively. These particles capture the protruding structure for the original and simulated data sets, where appropriate choices for particle diameter *d* yield an average of two particles per cell. Figure S9 in Supplementary Material illustrates how particles account for cell growth in biomovie D1: the elongation triggers an intermediate particle, and then cell division produces additional particles that track the new cells (independently of the strong noise and the cells in direct contact).

#### 5.2.2 Temporal Coherence

Our use of particle tracking to link particles into trajectories eliminates spurious phenomena while capturing temporal coherence within the biomovie. Figure S10 in Supplementary Material compares the two different time intervals of 5 frames (A) and 3 frames (B) for biomovie D3 where 38 versus 31 particles are filtered, respectively. These results are characteristics of our sensitivity analysis, which shows that the algorithm is robust to small changes in this parameter, even if setting larger time windows results in fewer particle trajectories. Figure S11 in Supplementary Material shows particle linking over 25 frames from a single simulated movie, where 383 particle positions were detected, resulting in 63 unique trajectories after linking, reducing this number to 34 unique trajectories after time filtering.

#### 5.2.3 Computational Performance

Nine data sets are shown in Figure S12 in Supplementary Material. The smallest data took between 25 and 39 s, and the largest between 1 min 30 s and 6 min. The average processing time roughly corresponds to the density of the biomovie’s cells, more so than simply the number of frames. The longest computation of 6 min was that of the particular case: a very densely populated colony (DS4 with ~1,700 cells). We chose a particle diameter of 7 px, with a minimum cell diameter of 17 px, which allowed us to identify and track 7,661 particles. We then chose a small temporal filtering window of 5 frames. After the time filtering step, 95% of 7,661 particles were removed. This example demonstrates the need to adjust the user settable parameters appropriately for the biomovie data set on a case-by-case basis.

#### 5.2.4 Summary

The particle detection and particle trajectory construction step successfully captures the spatial and temporal information in the binary image sequence without having to compute an explicit image segmentation at the level of individual cells. Our approach is computationally efficient and requires no manual intervention. It resists to transient interactions between neighboring cells that could lead to poor segmentation results in individual cell detection attempts.

### 5.3 Patch Lineage Graphs

**Figure 6:**
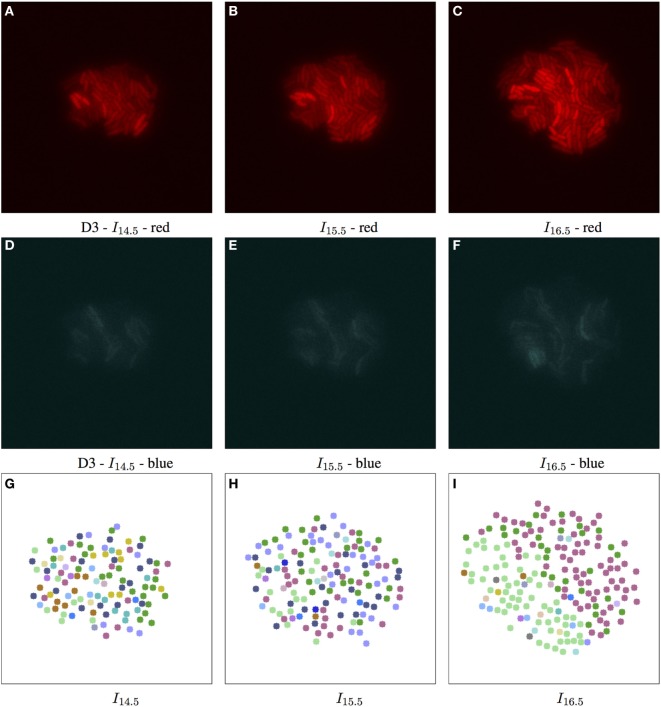
Biomovie D3 with RGB channels of image points 14.5, 15.5, and 16.5 h, and their corresponding patch structure, respectively. Enhanced exposures for red: 60% and blue: 90%. The S. meliloti bacterial cells are bio-engineered to fluoresce in a particular way, where each channel encodes a certain trait, or behavior. The red **(A–C)** and blue channels **(D–F)** show certain behavior in response to changes of conditions; here the bacterial cells are of wild type, and exposed to high concentrations of phosphate, influencing bacterial communication. The green channel is omitted due to its homogenous fluorescence. The patch structure is found using the following thresholds: geometric distance 100 px, and specific channel differences of red: 20, green: 50, and blue: 50. Main images show 7-px dots at computed particle locations. **(G–I)** The split/merge computation has been run, and particles are colored by their patch ID.

**Figure 7:**
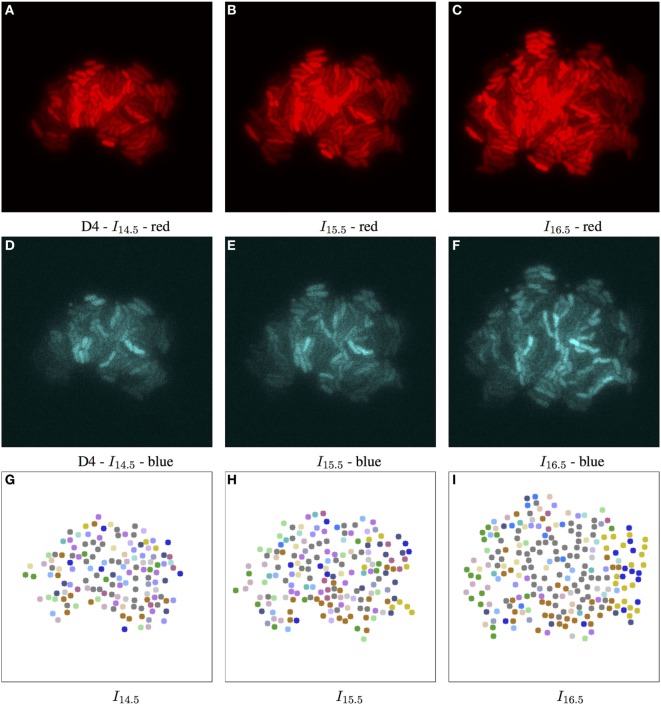
Biomovie D4 with RGB channels of image points 14.5, 15.5, and 16.5 h, and their corresponding patch structure, respectively. Enhanced exposures for red: 60% and blue channels: 90%. As seen in Figure 3, the biomovie showcases bio-engineered S. meliloti bacterial cells fluorescing in a particular way: The red **(A–C)** and blue channels **(D–F)** show certain behavior in response to changes of conditions; here the bacterial cells are of wild type, and exposed to high concentrations of phosphate, influencing bacterial communication. The green channel is omitted due to its homogenous fluorescence. The patch structure is found using the following thresholds: geometric distance 100 px, and specific channel differences of red: 20, green: 50, and blue: 50. Main images show 7-px dots at computed particle locations. **(G–I)** The split/merge computation has been run, and particles are colored by their patch ID.

Adjusting the thresholds of our approach allows us to focus individually on the spatial and/or temporal coherence of features. Figure 4 shows some interesting combinations determined through empirical experimentation, where the complex structure of biomovie D3 is revealed with high variation across the three channels (top row: A–C) of red, green, and blue. This variation is captured in three alternative patch lineages annotated atop binary images (Figures 4D–F). It also shows two examples from our sensitivity analysis benchmarks (Figures 4G,H), where only one channel is investigated while the others are ignored, for example, for the red channel: **v***_t,p_* = (*r_t,p_*), i.e., close to the image size for geometric distance and near the maximum of 255 for color channels. Figure 6 and Figure S14 in Supplementary Material further illustrate the implications of complex spatial and multichannel structure of biomovies. They depict patch assignments before and after the split/merge phase of the computation for biomovie D3. By comparing the patch structure to the fluorescence pattern in the RGB images, Figures 6 and 7 show consistent spatial and temporal assignments. This can be seen by looking at location and color of the patches in (L) relative to the spatial distribution and the variation of the fluorescence signal through the image space in (C). This signal varies from weak to moderate to high fluorescence, as seen in the center and through the colony. Temporal coherence refers to the consistency of the patches’ color throughout time. It is present in both D3 and D4, but the results of the D4 biomovie in Figure 7 depict not only a color consistency but also a spatially structured organization of patches in (J–L). We have also carefully validated the algorithm on the DS5 simulated movie. This verification is designed to allow the naked eye to check the correct patch structure. Supporting Figure 8 shows a sequence highlighting the behavior of the split/merge phases of the algorithm for this biomovie.

**Figure 8:**
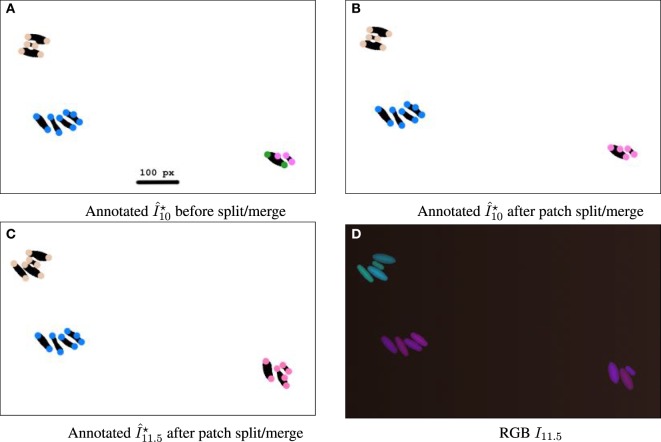
Sequence illustrating the split/merge computation with simulated movie DS5, designed to allow patches to be verifiable by the naked eye from the RGB image. Simulated movie DS5 is available from the study by Wiesmann et al. (2017). Images are cropped to a 787 × 482 px subset. **(A–C)** Binary images are annotated with colored circles, 16 px wide. The color encodes the patch ID. The geometric distance threshold for patch construction is set stringently to 100 px. **(A)** At time 10 h, before split/merge computation, showing four current patches. The bottom right quadrant has two neighboring cells with differently colored particles showing current assignments to different patches. **(B)** After split/merge computation, the particles are indeed the same color, showing that the patches have been merged as the patches are within the threshold distance to each other and have similar fluorescence. **(C)** At time 11.5 h, both the top left patch and the bottom right patch have new cells, and after the split/merge procedure is run for this time point they have correctly been assigned to the correct patch. **(D)** RGB image at time 11.5 h.

#### 5.3.1 Computational Performance

Our benchmarks show that the time required to create patch trajectories and patch lineage graphs primarily varies mainly depending on user-adjustable thresholds for geometric and color channel distances that define patch boundaries. The use of feature thresholds that favor aggregation into fewer patches allows faster computation. On the contrary, adjusting these thresholds to create a fine-grained structure of many patches increases the time spent computing splits and merges.

#### 5.3.2 Summary

We are demonstrating an application of CYCASP for colony-scale extraction of lineages of over 300 cells with automatic methods for the first time. Our patch lineage construction algorithm aggregates and simplifies the spatial-temporal changes that occur in a biomovie into a unified data structure, with a small number of parameters that can be set to control the level of detail represented. The multi-propagation algorithm works both forwards and backwards over time. It takes advantage of knowledge on the last time point of a biomovie to reap deeper benefits from temporal coherence than previously proposed methods.

### 5.4 Biological Interpretation

In E2, we were able to identify different subpopulations^1^ under similar conditions in D3 and D4. High phosphate concentrations in the medium interfere with cellular communication by repressing quorum sensing signaling, resulting in modified fluorescence signals. In this state of stress, we have found subpopulations that adapted to such levels by setting user thresholds to favor variation in the red channel. In biomovies D3 and D4, splitting and merging of patches revealed regions that showed changes in the activity of the reporter genes, which indicated a switch in cell state. In Figure 6, the patch structure represents a clear delineation of three main patches at time point 16.5 h. This suggests that the colony has developed into coherent subpopulations, which may either be the result of a stochastic event or an adaptive event to changes in the medium.

To study the D4 biomovie and find similarities or differences in colony growth, we used the same thresholds for the algorithm. Compared to Figure 6, we observe more patches in Figure 7, where subpopulations developed into different local regions of the colony. This suggests a more important disruption of bacterial colony growth, but triggered other cells to enter the quorum sensing state. For both biomovies, we observed a homogenous activity of the mVenus gene reporter in the green channel where the yellow fluorescence is distributed homogeneously between the bacterial cells. This indicates that the older the colony, the higher the quorum sensing signal. As can be seen in the blue channel (mCerulean), the heterogeneous activity of the exopolysaccharide gene reporter is captured by the results of the patch lineage.

In addition, based on the number of particles at time point 16.5 h and throughout biomovie D4, the colony grew faster than expected. For D3 and D4, we found at time point 16.5:253 versus 356 particles, and 5,679 versus 8,207 particles, respectively. This suggests that the D4 colony grew 1.4 times faster than the D3 colony. By setting the same thresholds for the two biomovies and promoting variation in the red channel, we have seen that both colonies are also able to adapt to changes in the environment. It is reflected by the spatial coherence or the structure of the patches found in Figures 6L and 7L. Temporal coherence is demonstrated by the consistency of patch color over time in Figures 6J–L and 7J–L. Moreover, compared to D3, the results of the D4 biomovie patch lineage show a structured spatial organization of the patches over the different time points (J–L). However, they present a rather fragmented view of the colony, suggesting that the growth of a dozen or so subpopulations occurred in the early stages of colony growth. We hypothesize that these subpopulations are the result of stochastic events that distinguish this biomovie. Using the patch concept, we find subpopulations, find differences between data sets, and follow the diversity and how quickly colonies grew in biomovies. The same methodology has been successfully applied to D1 and D2, which is also reflected in their analysis. They highlight the biological heterogeneity of population growth, where the two resulting colonies present distinct and non-uniform fluorescence signals, resulting in an even greater number of patches. A detailed discussion of these data sets is beyond the scope of this paper.

The concept of the patch lineage graph is biologically motivated: automatically computed graphs are intended to help microbiologists understand how and when changes in cell state occur in microbial populations. The patches, i.e., contiguous regions bounded by similar fluorescence patterns, provide insight into the development of bacterial cell colonies. In addition, the particle abstraction that we have proposed allows us to successfully treat cell division and exponential bacterial growth.

The simulated movies used in this article have been designed as minimal working examples that serve as understandable examples for testing and illustrating the CYCASP algorithm. They contain objects that mimic cell morphology and, to some extent, cell behavior. Biomovies demonstrate the response of bacterial colonies to experimental disruptions relative to normal development for both experiments (D1, D2) and (D3, D4).

## 6 Discussion

The CYCASP framework addresses the fivefold challenge of high cell count, high cell density, high cell shape diversity, strong noise, and high resolution by using the abstractions of particles, patches, and the DAG patch lineage. Our results are the first automatic solution to the problem of efficient comparative analysis of an arbitrary number of biomovies. Since a complete manual annotation of a biomovie can last one to two full working days, a computer-based approach that evaluates particles and patches instead of single cells can provide at least one valuable and additional view of the data. It can even provide an alternative to overcome the bottleneck of analysis. Our proposed abstractions succeed in exploiting and qualitatively integrating spatial and temporal coherence without explicit segmentation at the cellular level. It has been shown that the particle and patch have succeeded on biological data sets (i.e., biomovies) and simulated movies.

We have shown the effectiveness of the particle abstraction in handling complex biomovies targeted in this paper, where there is a daunting combination of high cell density, exponential growth, and a low temporal resolution. For other experimental setups (e.g., Petri dish) and different resolutions, CYCASP is generalizable but would require fine-tuning of parameters to handle specific scenarios (e.g., overlapping cells, where *W*_max_ should be set to compensate for the loss of particles).

Our framework is the first attempt to study subpopulations at the biological level of organization of cell groups that share a common ancestry. We call them patches that have similar behavior. From a certain point of view, our approach relates to the clustering problem definition. Indeed, it relies on some kind of clustering process, where particles are grouped into patches according to different criteria: (a) particle trajectories into patch trajectories using tracking information, (b) similarity in fluorescence characteristics, and (c) spatial closeness. This implies that members of one patch are more similar to each other regarding space and fluorescence features compared to other patches, other members of other patches are as well. Therefore, on a high level, we achieve a grouping of particles which is a clustering. Yet on a low level, we would not call it a clustering algorithm so not to cause confusion. Moreover, clustering usually refers to joining or grouping entities according to some similarity or distance measure defined in the feature space of the similarities (which covers vector quantization, agglomerative, and divisive clustering methods).

Our results show that patches and patch trajectories are an intuitive, flexible, and powerful concept. They reflect different cellular behaviors for subpopulations that split off from each other at certain times and merge together in others. Our modular and automatic patch lineage algorithm has succeeded in constructing patch trajectories on all time points of the biomovie. With appropriate parameter settings, these trajectories can be assembled into a patch lineage DAG that captures the high-level behavior of interest. As we mentioned in the Methods section, the computational efficiency of our framework hinges on processing far fewer image objects in far more depth at each stage. However, in light of the properties of the above-mentioned data, particle identification is much more effective than the detection of individual cells. Our collaborators directed the manual analysis and reconstructed very low level views of the ancestral relations between the cells. Their underlying motivation was to better understand the high level behavior of a colony. We maintain that our framework allows us to achieve this goal. The innovation with CYCASP is to support this level of analysis directly and automatically.

CYCASP succeeds where previous automatic methods fail because we avoid the bottleneck of having to perform a segmentation for each individual cell. In addition, a patch lineage DAG has a much simpler structure than a cell lineage tree because it has far fewer branches. This simplified abstraction is designed to help our collaborators directly understand and reason about the behavior of entire cell colonies at the biologically relevant level of cell subpopulations with similar behavior, rather than infer it from the overwhelmingly complex branching structure of individual cell lineages. Moreover, in other knowledge domains such as stem cell research, interest shifts to subpopulations where researchers are unable to distinguish between an early stochastic event and the existence of a predetermined subset of cells that are in some way primed for cellular reprogramming (Smith et al., 2010). This change in the general paradigm is motivated by the realization that is impossible to trace the origin of a subset of a colony or particular cells that detach from other colonies. In return, the methodology presented could address these biological issues. This article sets the stage for an easier way to analyze biomovies starring nanoscale organisms.

## Author Contributions

GH designed the framework, directed its development and contributed to data analysis, the drafting of initial manuscript and the revision of the manuscript. VW created the simulated data. AB interpreted the biological data and contributed to the revision of the manuscript. TM and TWN, contributed to framework development, manuscript writing and manuscript revisions. All authors read and approved the final manuscript.

## Conflict of Interest Statement

The authors state that the research was conducted in the absence of any commercial or financial relationships that could be interpreted as a potential conflict of interest.

## Footnotes

^1^The cells of the same subpopulation could be dispersed throughout the colony. Please note that this term is used in a special context to designate the cell patches detected by our algorithm that can represent cell subpopulations in a classical sense. With appropriate user thresholds, its usage is valid for our approach.
