# Supplementary material for: A Novel Methodology for Characterizing Cell Subpopulations in Automated Time-lapse Microscopy
Source: Front Bioeng Biotechnol. 2018 Feb 28;6:17. doi: 10.3389/fbioe.2018.00017 (PMC5835524; doi:10.3389/fbioe.2018.00017)

Supplementary Material
'A novel methodology for characterizing cell sub-populations in automated time-lapse microscopy'

Georges Hattab, Veit Wiesmann, Anke Becker,
Tamara Munzner, and Tim W. Nattkemper

S1 Table. Specifications for the 4 biological and 5 simulated movie data sets used in this paper.

Specifications	Biomovies	Simulated movies
datasets	4 sets (D1–D4)	5 sets (DS1–DS5)
channels	RGB	4 sets green-only, 1 set RGB (DS5)
frame count	N = 115 (D1, D2), 44 (D3, D4)	N = 25, 60, 63, 78, 76
hours of recording	57.5h (D1, D2), 22h (D3, D4)	varying times
lateral resolution	60 nm/px (high)	varying (low - moderate)
experiments	2 experiments w/ 2 conditions each	5 simulations
cell organism	*S. meliloti (in situ)*	cell model *(in silico)*
cell count	∼300 cells (D1, D2), 80 cells (D3, D4)	vary from ∼70 to ∼400 cells
cell shape diversity	high variation (from rod-shape to contiguous cells)	low variation (elliptical or oval)
cell shape size	high variation	no variation
cell density	high density	high density
cells in contact	touching cells (no overlay)	touching with few overlays, no touching

S1 Appendix. Simulated movies. Simulated movies were created by employing: cell simulation, shape, texture, channels with noise and artifacts. While the cell simulation software (Wiesmann et al. 2013) has been extended for biomovie simulation, the steps for simulation are similar to image simulation. First, the cell shape is calculated. Second, the cell position on the image. In the third step, the cell texture is added. In the fourth and last step, imaging artifacts and noise are added. For the simulated movies, bacterial shapes are modeled as ellipses with varying length of semi-major and semi-minor axis. Bacterial cell positions are determined on a frame by frame basis by minimizing an energy function. For the first frame the first bacterium is placed in the image centre. After it has divided, the new bacterium is placed next to the bacterium of which it originates from. After all bacteria for the frame have been calculated, the bacteria are input to the following energy equation:

$$E^*(bacteria) = \sum_{o}^{N_o} \sum_{p \in o} I_{dist}(p) + k * \sum_{o_1 \neq o_2}^{N_o} \sum_{p_1 \in o1} \sum_{p_2 \in o2} \delta(p_1, p_2) \tag{1}$$

Where:

N_o = number of bacteria
I_{dist} = distance transformed mask of the bacterial cell shape
$\delta(p_1, p_2)$ = equal to 1 if the below condition is fulfilled.

If $p_1 == p_2$ pixels of bacterium o_1 and o_2, respectively. Else, $\delta(p_1, p_2) = 0$.

The first energy summand keeps bacteria sticking together in the image centre. The second energy summand prevents overlap between bacteria. The factor k weighs energy summand one against the second energy summand. The gradient descent method is applied to iteratively minimize the energy equation to find the positions of bacterial cells on the current frame. Their positions at the previous frame are the starting point for energy minimisation at the current frame. Three channels are simulated with varying appearance modelling the properties of various real fluorophores. The bacteria texture is calculated with the sigmoid function as written below.

$$f_{sigmoid} = \frac{I_i}{1 + e^{\kappa * v}} \tag{2}$$

I_i = maximum intensity of the texture of bacterium i (Gaussian distributed)
v = distance transformation value for the corresponding pixel on the mask
κ = controls the slope of the intensity at the bacteria edges

The bacterial intensity is highest in blue channel with lowest variability. The green channel has medium intensity level, and variability. The red channel has the lowest intensity, and the highest variability. Each channel depicts linearly increasing background intensity from the left to the right side of a modeled cell to simulate illumination inhomogeneity. This slope of the intensity ramp is chosen to be increasing from blue channel over the green channel to the blue channel. Gaussian noise is added with increasing level from blue channel over the green channel to the red channel.

S1 Fig. Comparative illustration of the single-cell segmentation approach to our particle-based solution for constructing lineages in biomovies. (A) Single-cell segmentation is used to track object centroids, detecting cell mitosis explicitly, and constructing cell lineages accordingly. (B) Multiple particles are detected within regions, and tracked over time, detecting mitosis implicitly.

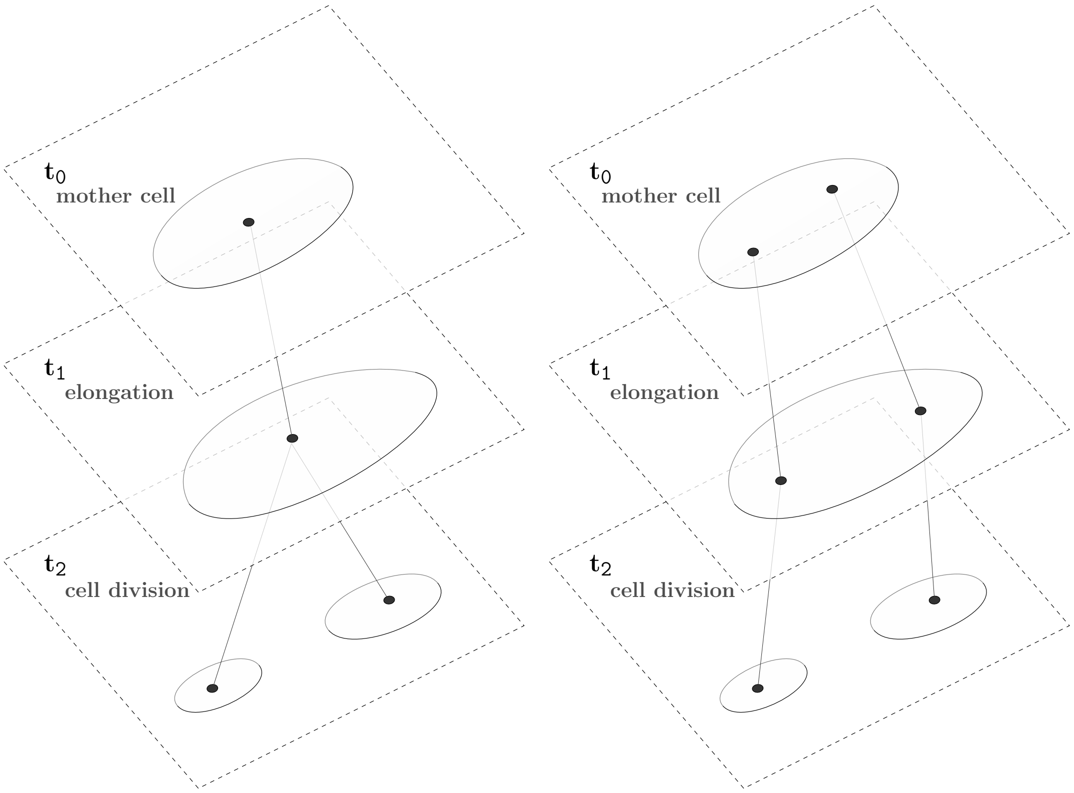

(A) Single-cell segmentation (centroids) (B) CYCASP approach (particles)

S2 Fig. Overview of the three steps of the CYCASP architecture. It comprises: preprocessing, particles, and patch lineages. Their respective function calls in the publicly available source code are referenced on the right side of the figure. The modular patch lineage algorithm uses the create, and propagate patches functions within its three internal stages: find patches, split patches, and merge patches.

Preprocessing
Input: RGB images
Output: binary images

For each time (frame) t from $0 \rightarrow t_{\max}$: `preprocess()`
enhance the signal to noise ratio
subtract and enhance local signals
adaptive background masking

Particles
Input: binary images
Output: particle trajectories

For each `get_data()`
time point: particle finding, and tracking
trajectory: trajectory linking
trajectory: trajectory time filtering
particle: particle color re-adding
trajectory: trajectory color filtering

Patch Lineages
Input: particle trajectories
Output: patch lineages graph (.gml or .json)

At time $t_{\max}$: `modalgo()`
1 - find patches
    2 - create patches
    3 - propagate patches

For each time t from $t_{\max} \rightarrow 0$
1 - find patches
    2 - create patches
    3 - propagate patches
    4 - split patches
        2 - create patches

For each time t from $0 \rightarrow t_{\max}$
  5 - merge patches
    2 - create patches
    3 - propagate patches

S2 Appendix. Preprocessing A pipeline of standard computer vision methods to reduce noise and enhance the object-to-background contrast.

1. Enhancing the signal to noise ratio (SNR)
   (a) RGB to greyscale transformation
   (b) image inversion
   (c) contrast limited adaptive histogram equalization (CLAHE) using a tile size $\tau = 3^2$ px, and contrast limit of 3, to clip, and uniformly distribute any histogram bin above that limit
   (d) pixel intensities transformation for a global contrast enhancement using the following formula: $I'_t = \frac{L}{\phi} \times (\hat{I}_t \times \frac{1}{L/\theta})^2$ with maximum intensity L=255, and $\phi = \theta = 1$ (Fig. S3(D)).
2. Subtracting and enhancing local signals
   (a) denoise bilateral filtering with spatial closeness $s_{\text{spatial}} = 75$, radiometric similarity $s_{\text{range}} = 75$, and pixel neighborhood size $\delta = 5$ px of each pixel neighborhood that is used during filtering
   (b) adaptive mean thresholding with block size $\tau = 13^2$ px, and constant $\mathrm{C} = 2$, that is subtracted from the weighted mean in order to prevent noise to pop up at background regions (Fig. S3(F)).
3. Adaptive background masking
   (a) median blurring with an aperture linear size $k = 15^2$ px
   (b) binary thresholding with $h = 255$, and maximum value $V_{\max} = 255$
   (c) masking, by using a binary mask of image dimensions $(r \times c)$ is initialized, containing the background. A bitwise comparison (disjunction) returns the foreground which contains the colony (Fig. S3(H)).

S3 Fig. Example results after each preprocessing step for original biomovie D1 at t = 57.5 h, the final frame. The RGB image is showcased here at 100% exposure, with close-up detail of the bottom left quadrant. (A) The input RGB image. (B) After the greyscale transfosrmation and image inversion. (C) After the contrast limited adaptive histogram equalization (CLAHE). (D) After the global contrast enhancement. (E) After the denoise bilateral filtering. (F) After the adaptive mean thresholding. (G) After the median blurring. (H) After masking, the final output is a binary image. For the detailed preprocessing, see supplemental Appendix S2.

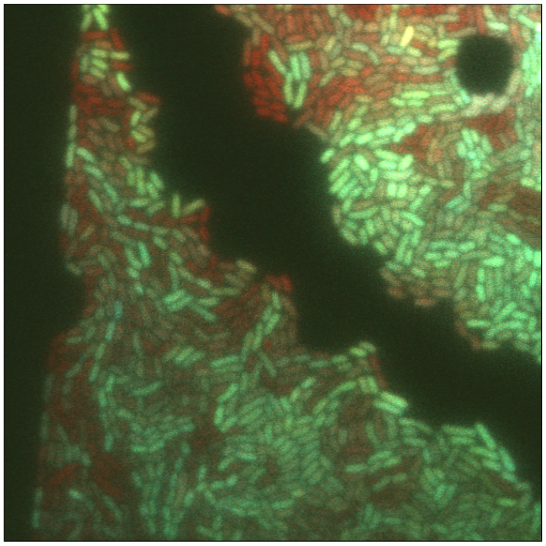

(A) input: RGB

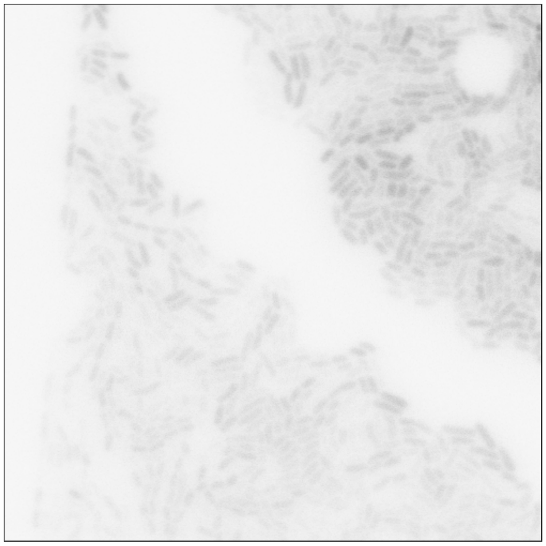

(B) greyscale, invert

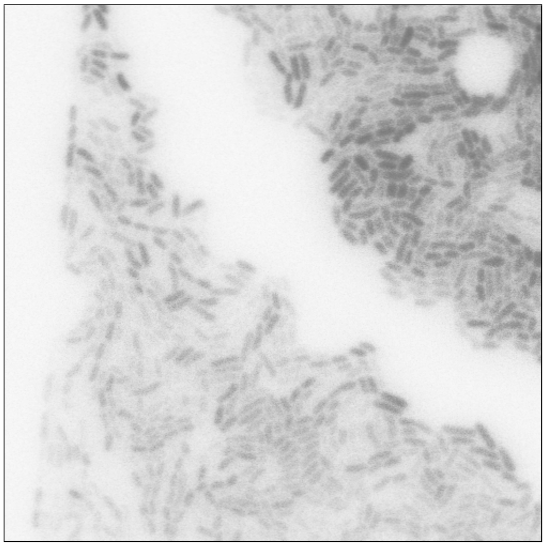

(C) CLAHE

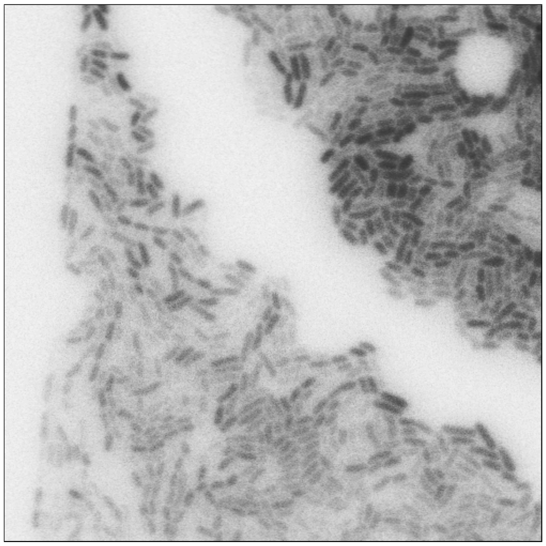

(D) global contrast

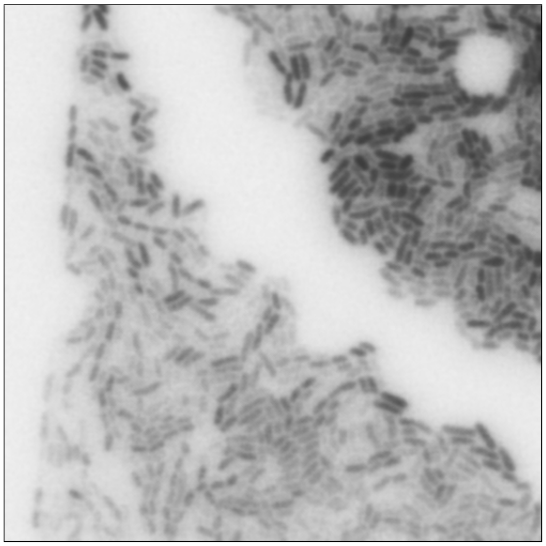

(E) denoise bilateral

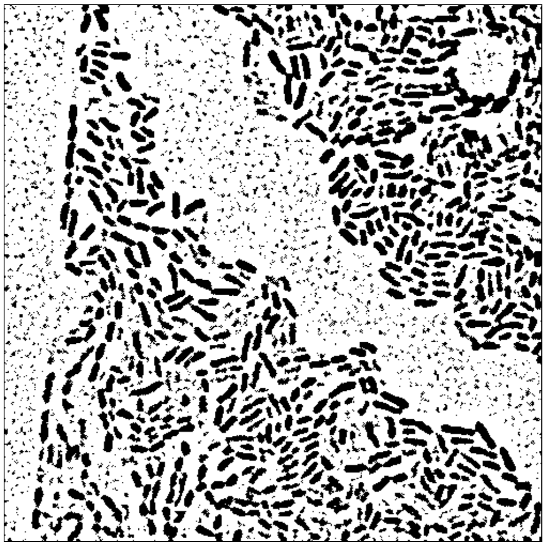

(F) adaptive threshold

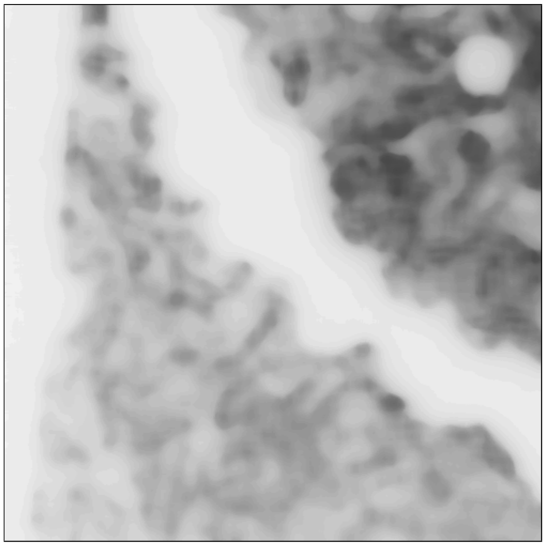

(G) median blur

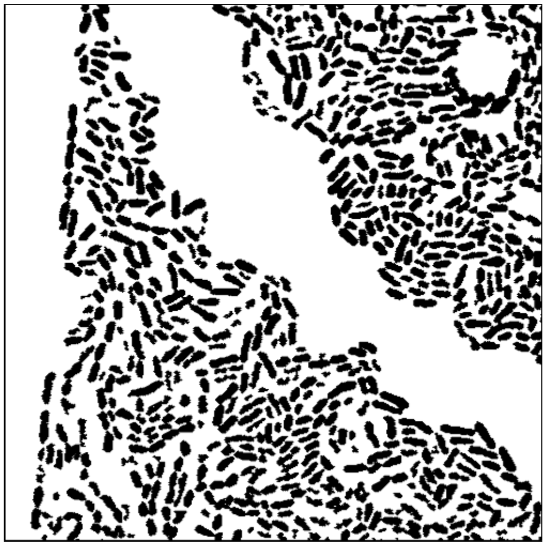

(H) output: binary image $\hat{I}_{115}$

S4 Fig. Binary images after preprocessing of the original biomovie final frames (D1–D4). (A) Biomovie D1 shows a phenotypic heterogeneity experiment, with two separate colonies visible. (B) Biomovie D2 is an alternate condition of the same experiment. (C) Biomovie D3 shows an experiment on bacterial communication by quorum sensing. (D) Biomovie D4 is an alternate condition of the same experiment.

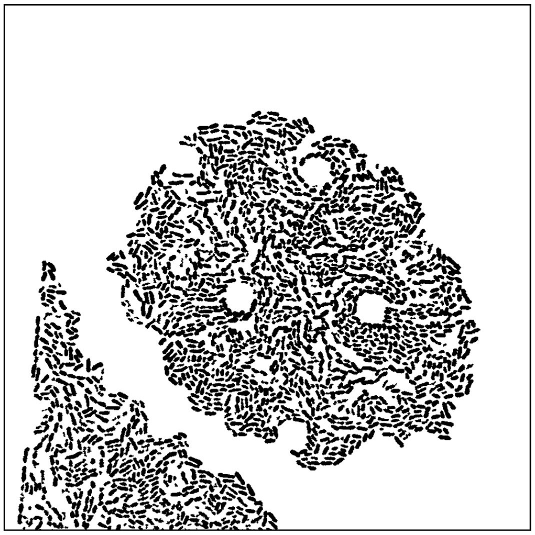

(A) D1 - $\hat{I}_{57.5}$

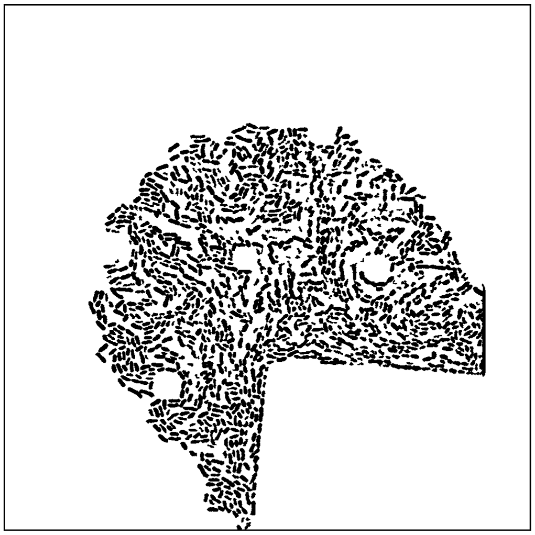

(B) D2 - $\hat{I}_{57.5}$

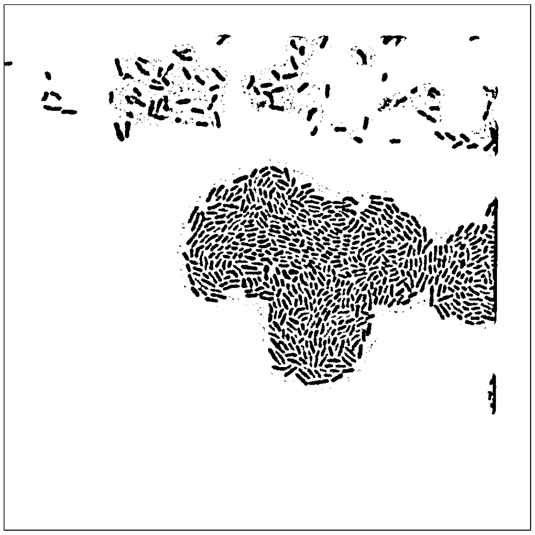

(C) Biomovie D3 - $\hat{I}_{22}$

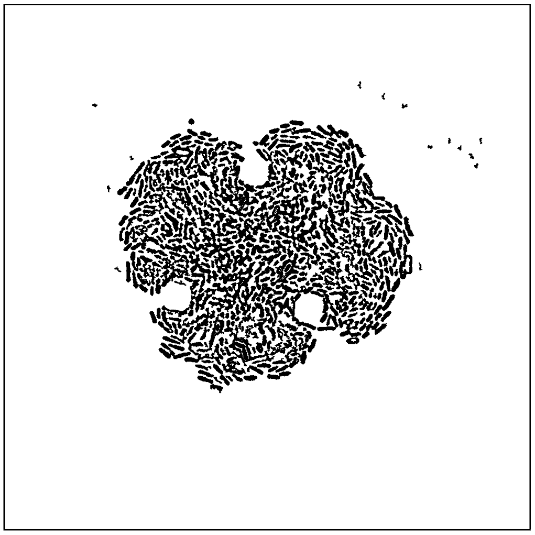

(D) D4 - $\hat{I}_{22}$

S5 Fig. RGB images of the final frames from simulated movies DS1, and DS2. (A) DS1. (B) DS2.

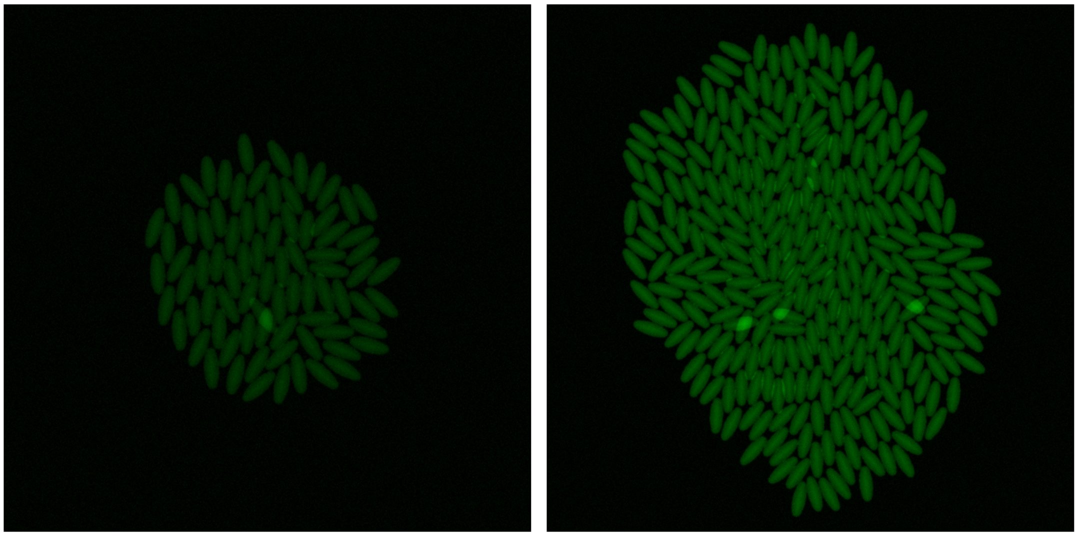

(A) DS1 - I_{25} (B) DS2 - I_{60}

S6 Fig. Binary images after preprocessing of RGB images in S5 Fig. (A) DS1. (B) DS2.

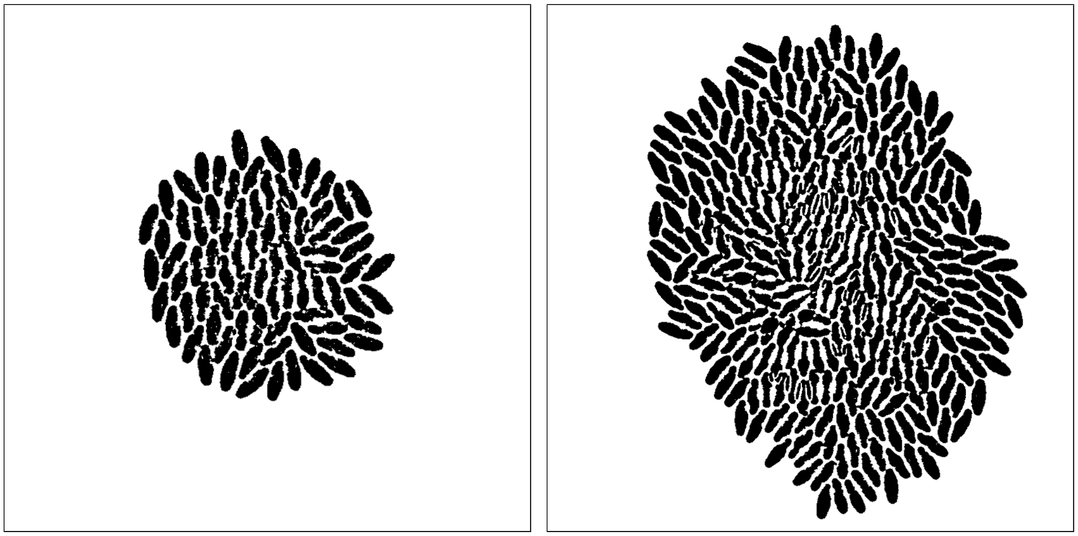

(A) DS1 - $\hat{I}_{25}$ (B) DS2 - $\hat{I}_{60}$

S7 Fig. RGB images of the finale frames from simulated movies DS3, and DS4. (A) DS3. (B) DS4.

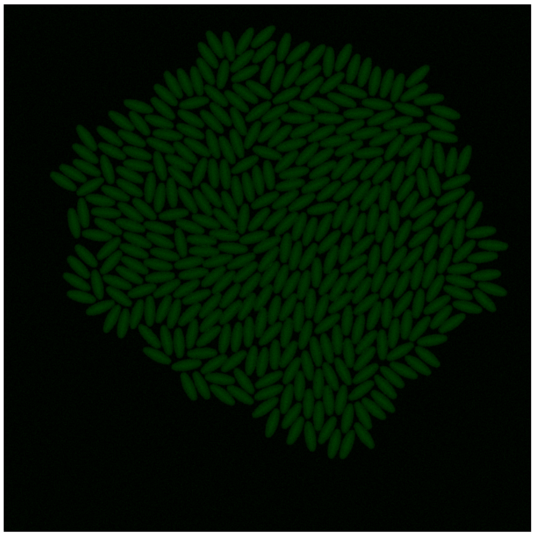

(A) DS3 - I_{63}

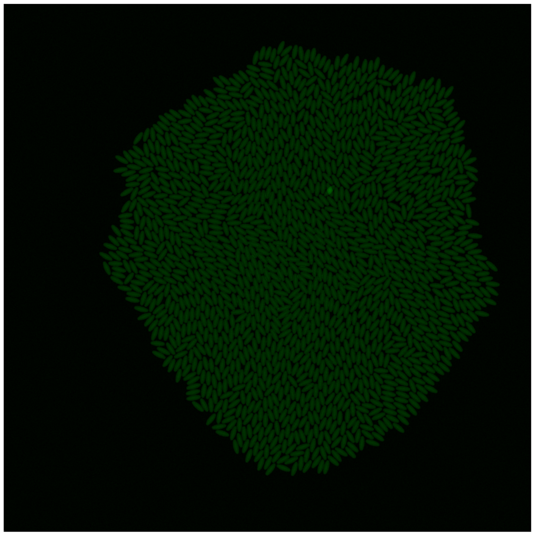

(B) DS4 - I_{78}

S8 Fig. Binary images after preprocessing of RGB images in S7 Fig. (A) DS3. (B) DS4.

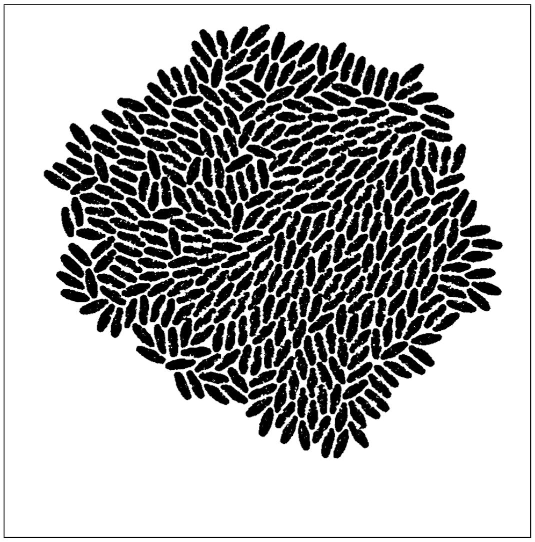

(A) DS3 - $\hat{I}_{63}$

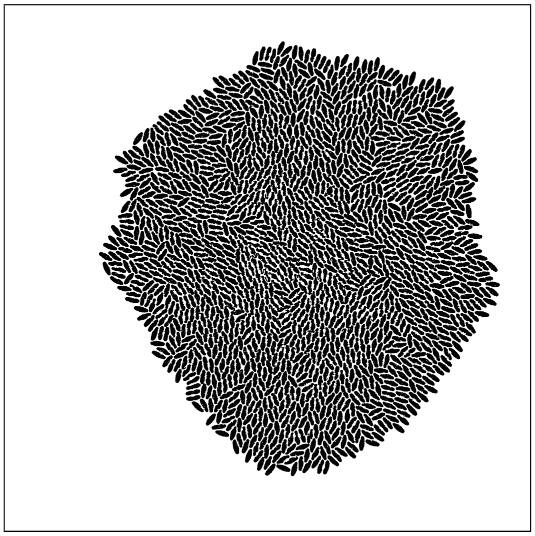

(B) DS4 - $\hat{I}_{78}$

S9 Fig. Particle detection for original biomovie D1 across cell division events. Detected particle locations are annotated as red circles on original images (A-C, G-H), and white circles on binary images (D-F, I-J). The particle paradigm handles cell division cleanly despite high levels of noise, and the direct contacts between cells: when the cell elongates, a new particle is created in the centre when the width between the previous particles surpasses the distance threshold.

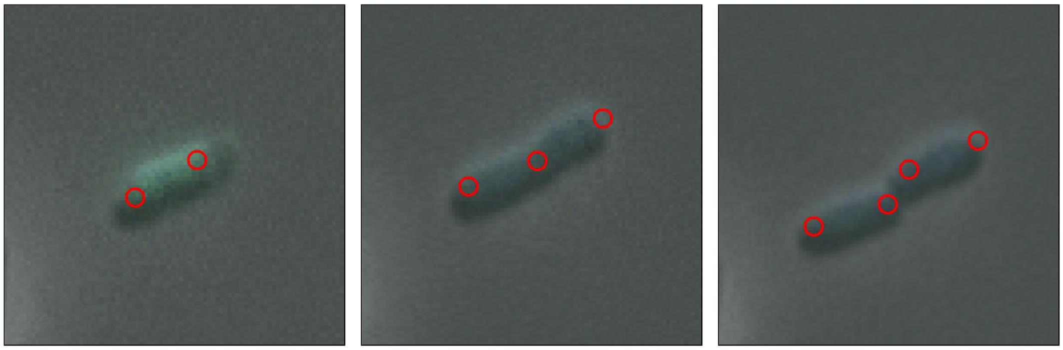

(A) RGB $I_{0.5}$ (B) RGB I_7 (C) RGB $I_{8.5}$

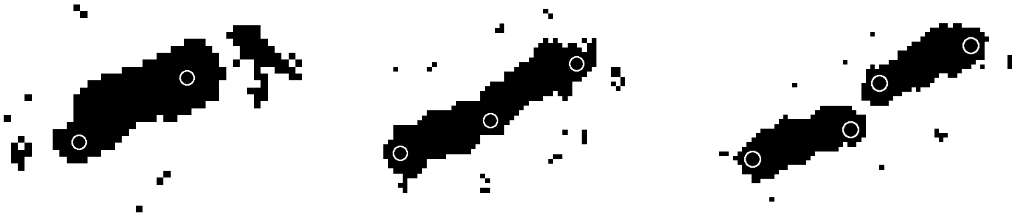

(D) Binary $\hat{I}_{0.5}$ (E) Binary $\hat{I}_7$ (F) Binary $\hat{I}_{8.5}$

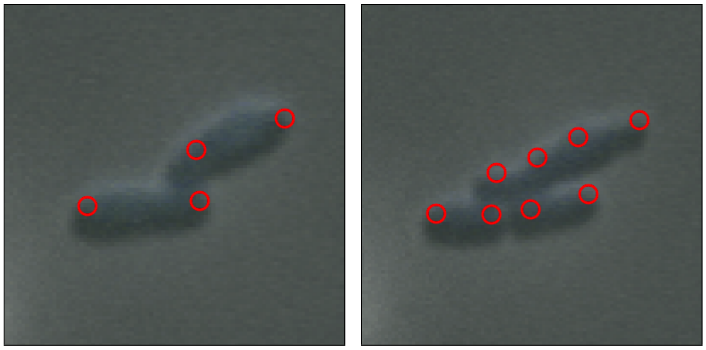

(G) RGB I_9 (H) RGB $I_{10.5}$

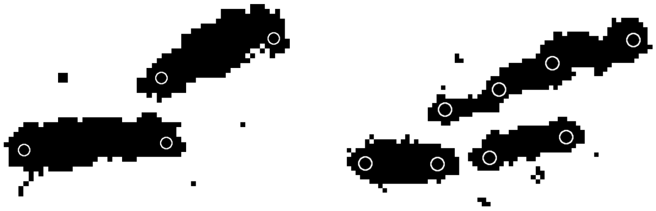

(I) Binary $\hat{I}_9$ (J) Binary $\hat{I}_{10.5}$

S10 Fig. Effect of the time filtering window on particle trajectories in biomovie D3. Binary images are annotated with eliminated particle positions with large 7-px magenta circles. (A) A 5-frame window filters out 38 particles. (B) A 3-frame window filters out 31 particles. Blue arrows highlight some of the particles kept for the shorter window but filtered out in the longer window.

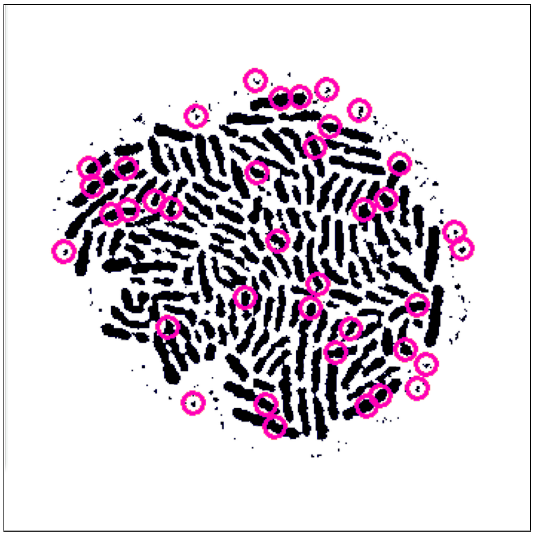

(A) (D3 $\hat{I}_{16.5}$) d= 9 px, time filter window 5 frames

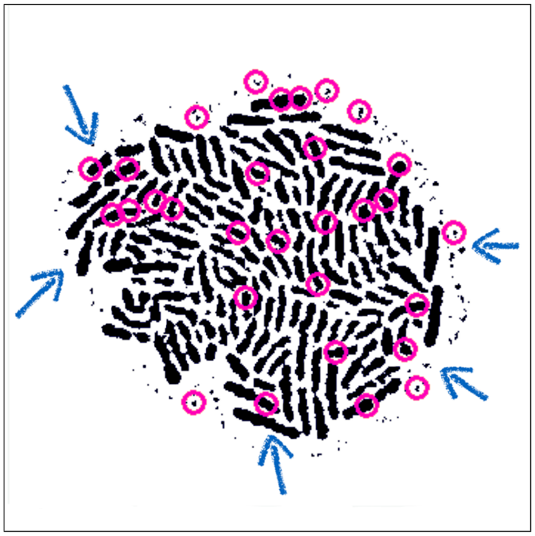

(B) (D3 $\hat{I}_{16.5}$) d= 9 px, time filter window 3 frames

S11 Fig. Example result of particle linking for simulated movie DS5. The result is shown for cropped 375x500 px subsets of the original 2048x2048 px images depicting four to seven cells appearing in: cyan (top), and magenta (bottom) in (A–C). The black background was replaced by white pixels to better notice the cells and the background noise. The threshold for particle finding was diameter $d = 13$ px and for particle linking the time filtering window was set to 3 frames. Computed particle locations annotated as 10 px white dots in (A–C). (A) Time point 1 (30 min or 0.5 h) shows two ancestor cells. (B) By time point 10 (5 h) both ancestors have divided once. (C) By time point 20 (10 h) the upper cyan colony has 3 cells, and the lower purple one has 4. (D) Particle trajectories covering the first 23 time points (11.5 h) are shown by color coding each particle differently according the unique ID of the computed particle trajectory. This image crop contains 19 unique trajectories, all of which show an overall downward drift. For the entire DS5 biomovie, we globally found 383 particle positions resulting in 63 unique trajectories after linking, reduced to 34 trajectories after time filtering.

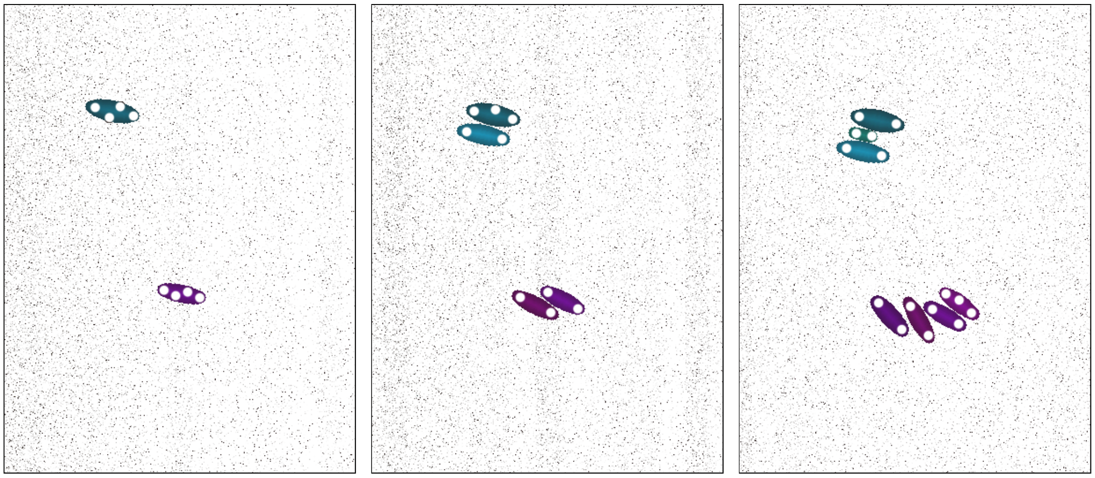

(A) RGB $I^{\star}_{0.5}$ (B) RGB $I^{\star}_{5}$ (C) RGB $I^{\star}_{10}$

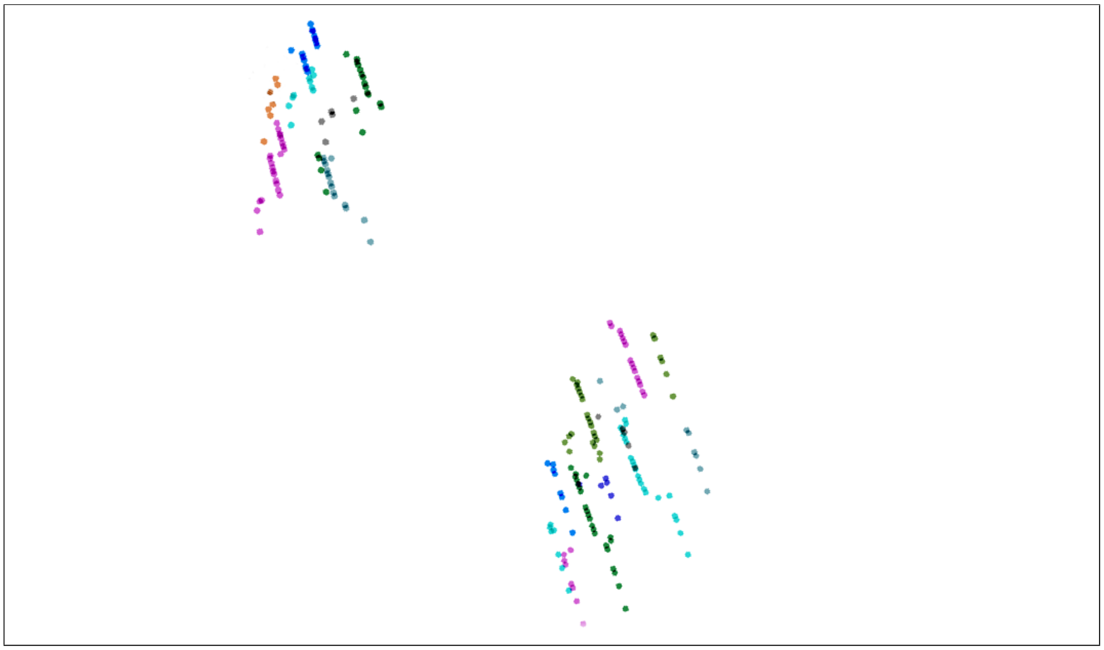

(D) Particle trajectories found across time: $t_0.5$–$t_{11.5}$

S12 Fig. Average elapsed time of 100 runs of the preprocessing step for all biomovies, in seconds. Biomovies in the x-axis are sorted by frame count, from lowest to highest (as indicated in parentheses). We observe an approximate correlation between frame count and preprocessing time. The average time varies with a $\Delta \pm 1$ second(s). A mid-2013 MacBook Air (1.7GHz dual-core Intel Core i7, 8Gb of 1600MHz memory) was employed for all benchmarks.

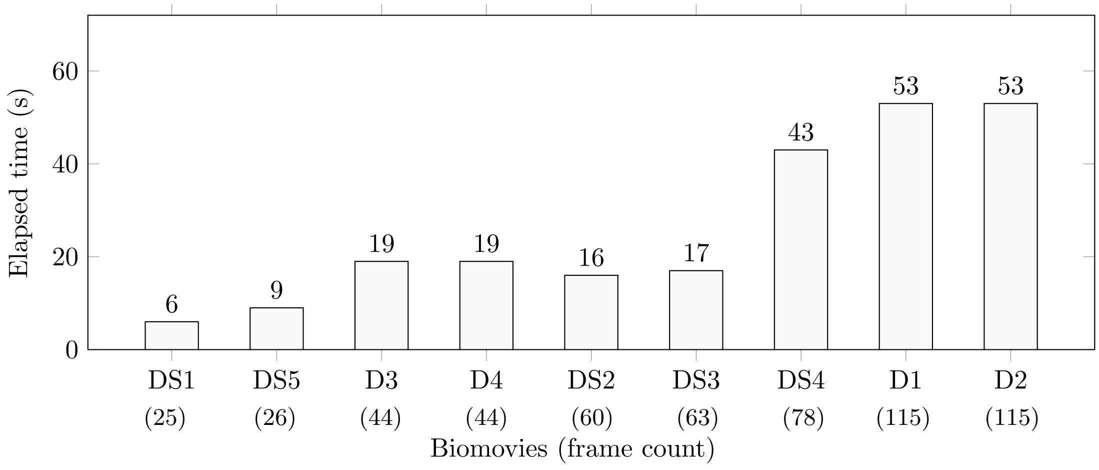

S13 Fig. Average elapsed time of 100 runs of the particle step for all biomovies, in seconds. The particle step includes all three phases of particle finding, linking and filtering. Biomovies in the x-axis are sorted by frame count, from lowest to highest (as indicated in parentheses). We observe that particle-related computation time is related to the density of the colony in the biomovie, rather than the number of frames. The DS4 biomovie is a highly dense special case, where finding and linking in time over 7000 particles takes over 6 min. The average time varies with a $\Delta \pm 2$ second(s). The particle step can be computationally expensive, given a highly populated colony and a particle diameter set to a low value.

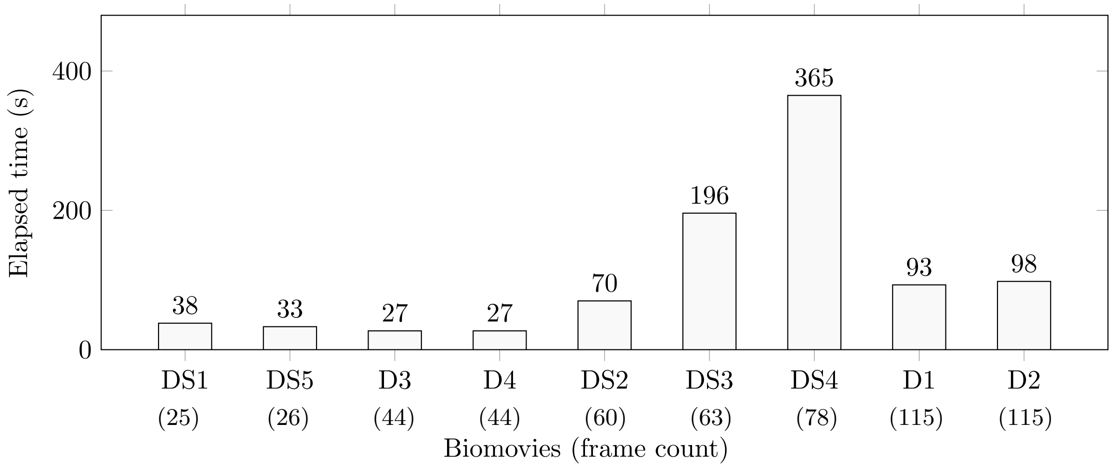

S14 Fig. Patch structure before and after splits/merges occur in biomovie D3 at time points 14.5 h, 15.5 h, and 16.5 h corresponding to the RGB images in Fig. 6. Thresholds are geometric distance 100 px and specific channel differences of red: 20, green: 50, and blue: 50. Main images show 7-px dots at computed particle locations; lower left corner shows original binary image. Top row shows three time points before the split/merge computation, and bottom row shows those time points after the computation. (A–C) Before the split/merge computation, all three time points have highly similar patch assignment patterns where particles are colored by their currently assigned patch ID. The initial patch creation computation has been run at the final time point (frame 44 in this case) and patch IDs have been propagated backwards to previous time points. (D–F) The split/merge computation has been run, and only particles with changed patch assignments are colored by their patch ID; unchanged particles with assignments matching the top row are grey. (D) Many colored new patch assignments reflect the fact that the previously propagated patch information was not valid at most of the particle positions for time point 14.5 h; splits and merges updated these assignments. (E) A moderate number of new patches reflect the difference in fluorescence patterns between the middle and right columns of the RGB images in Fig. 6, leading to updated assignments in the split/merge propagation. (F) Only a few singletons stand out with new assignment colors against the mostly grey points reflecting a globally unchanged patch pattern, showing that most trajectories visible at time point 16.5 h maintained correct assignments from the initial computation.

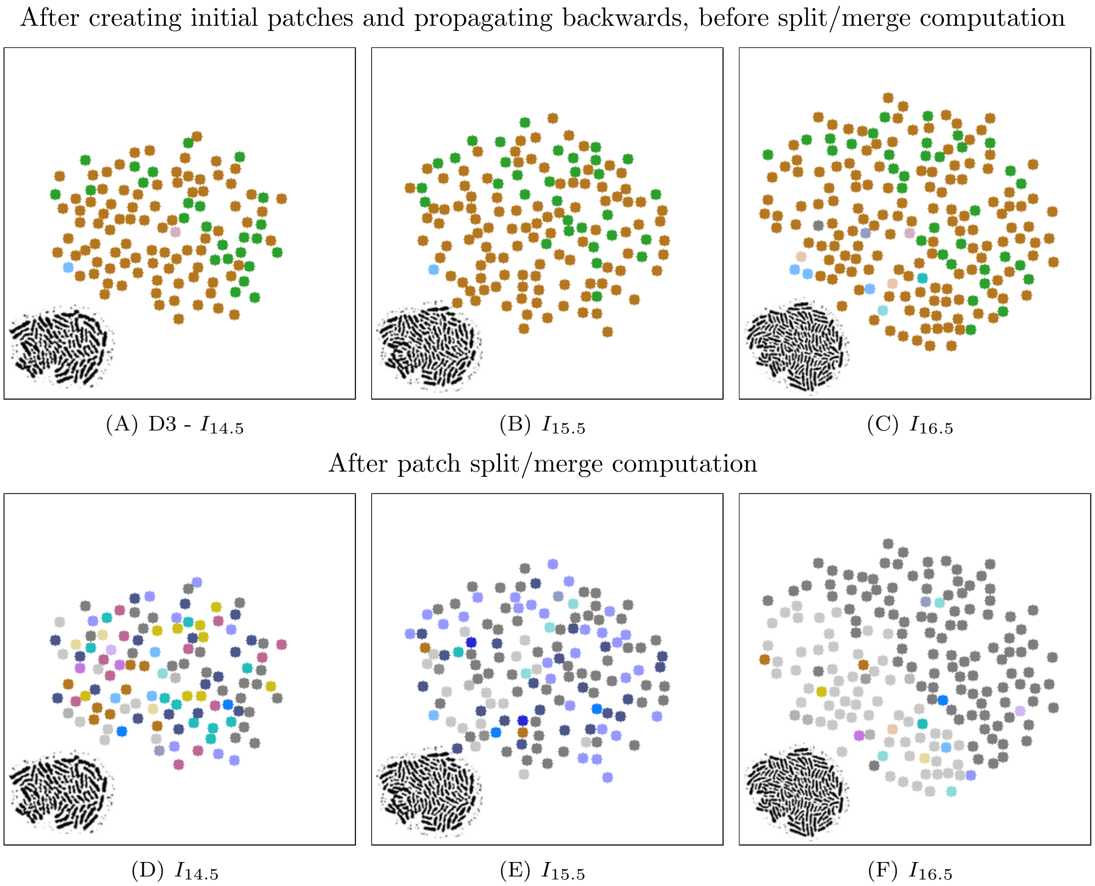

(A) D3 - $I_{14.5}$ (B) $I_{15.5}$ (C) $I_{16.5}$

(D) $I_{14.5}$ (E) $I_{15.5}$ (F) $I_{16.5}$

S15 Fig. Comparison of the number of annotated cells to the number of computed particles for four different time points for each biomovie. Observable cells were annotated using BIIGLE 2.0 (Langenkämper et al. 2017). The four time points in E1 were selected before the colonies grew out of the image space (i.e. D2) or right before another colony invaded the image space (i.e. D1). The employed parameters for both experiments are: $\sigma_{\max} = 7$ px, $W_{\max} = 5$ frames. The particle diameter for E1 and E2 is set to 7 px and 9 px, respectively. The particle trend is consistent per experiment. On average, we observe that there are at least 1.7 times more particles than there are cells. We calculated regression models based on the number of particles in each experiment: E1 based on 60 frames, and E2 on 40 frames. The trend fits to an exponential regression for both experiments. E1 results are consistent with the exponential trend in the first 21h of the biomovies as shown in Schlüter et al. 2015. We report the calculated regression parameter results and the average ratio of particles to annotated cells in the table below.

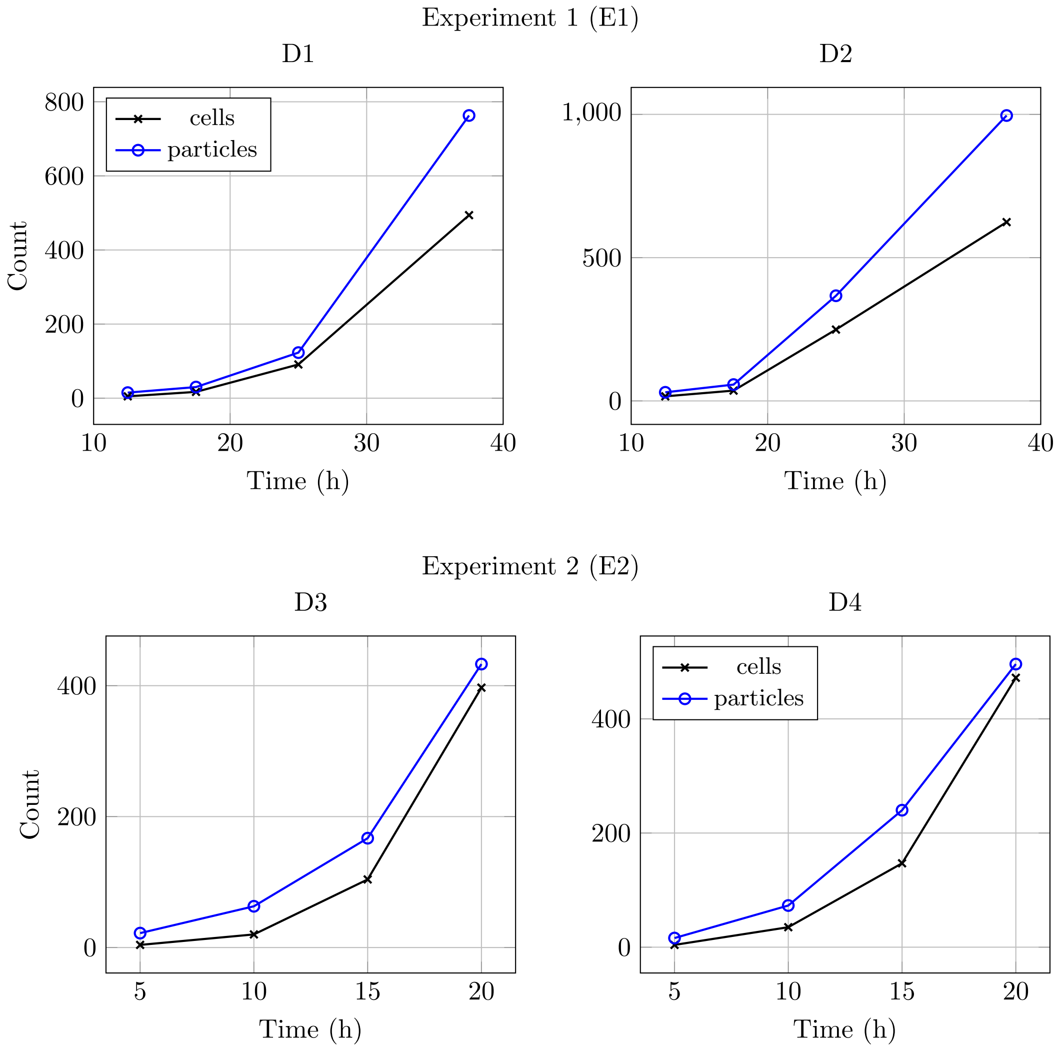

	correlation coefficient	coefficient of determination	average relative error*	average ratio of particles to annotated cells
D1	0.99	0.98	11.74	1.9
D2	0.96	0.93	14.23	1.7
D3	0.98	0.95	10.91	2.7
D4	0.99	0.98	26.03	2.2

*standard error of regression

S16 Fig. Intra-observer reliability for a five times manual annotation of a single image. A zoomed view of phase contrast image t_{25} from biomovie D2 is shown below. The phase contrast image was enhanced: contrast +40%, brightness +20%. Cell centroids are depicted as blue dots. The mean and standard deviation are $\mu = 267$ and $\sigma = 21.65$, respectively. The cells were annotated using BIIGLE 2.0 (Langenkämper et al. 2017).

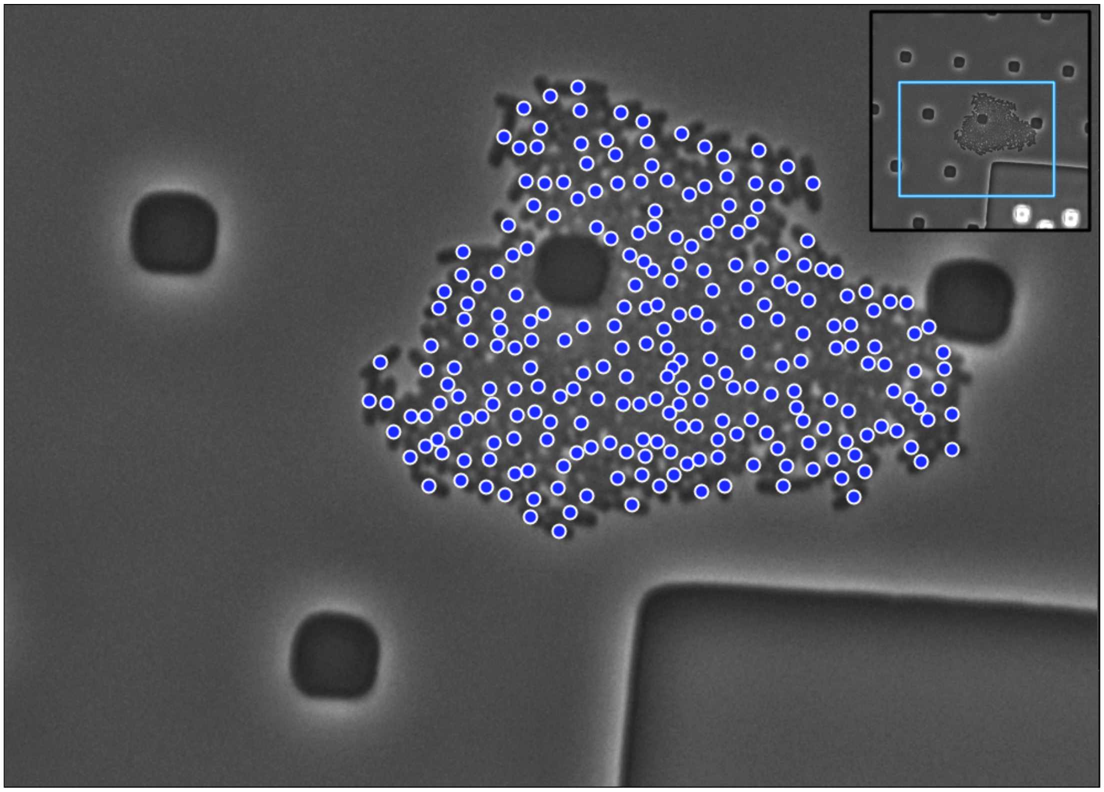

Supplement: Supplementary file 1 [file presentation_1.pdf]
